# Hyperoside alleviates toxicity of β-amyloid via endoplasmic reticulum-mitochondrial calcium signal transduction cascade in APP/PS1 double transgenic Alzheimer's disease mice

**DOI:** 10.1016/j.redox.2023.102637

**Published:** 2023-02-14

**Authors:** Lin Lin Song, Yuan Qing Qu, Yong Pei Tang, Xi Chen, Hang Hong Lo, Li Qun Qu, Yun Xiao Yun, Vincent Kam Wai Wong, Rui Long Zhang, Hui Miao Wang, Meng Han Liu, Wei Zhang, Hui Xia Zhang, Joyce Tsz Wai Chan, Cai Ren Wang, Jian Hui Wu, Betty Yuen Kwan Law

**Affiliations:** a Dr. Neher's Biophysics Laboratory for Innovative Drug Discovery, State Key Laboratory of Quality Research in Chinese Medicine, Macau University of Science and Technology, Macao; b Faculty of Chinese Medicine, Macau University of Science and Technology, Macao

**Keywords:** Alzheimer's disease, Aβ aggregates, Hyperoside, Neurotoxicity, Endoplasmic reticulum-mitochondrial-Ca^2+^, Ryanodine receptors, Calcium signal, Mitochondrial membrane potential

## Abstract

Alzheimer's disease is a neurodegenerative disorder characterized by a decline in cognitive function. The β-amyloid (Aβ) hypothesis suggests that Aβ peptides can spontaneously aggregate into β-fragment-containing oligomers and protofibrils, and this activation of the amyloid pathway alters Ca^2+^ signaling in neurons, leading to neurotoxicity and thus apoptosis of neuronal cells. In our study, a blood-brain barrier crossing flavonol glycoside hyperoside was identified with anti-Aβ aggregation, BACE inhibitory, and neuroprotective effect in cellular or APP/PSEN1 double transgenic Alzheimer's disease mice model. While our pharmacokinetic data confirmed that intranasal administration of hyperoside resulted in a higher bio-availability in mice brain, further *in vivo* studies revealed that it improved motor deficit, spatial memory and learning ability of APP/PSEN1 mice with reducing level of Aβ plaques and GFAP in the cortex and hippocampus. Bioinformatics, computational docking and *in vitro* assay results suggested that hyperoside bind to Aβ and interacted with ryanodine receptors, then regulated cellular apoptosis via endoplasmic reticulum-mitochondrial calcium (Ca^2+^) signaling pathway. Consistently, it was confirmed that hyperoside increased Bcl2, decreased Bax and cyto-c protein levels, and ameliorated neuronal cell death in both *in vitro* and *in vivo* model. By regulating Aβ-induced cell death via regulation on Ca^2+^ signaling cascade and mitochondrial membrane potential, our study suggested that hyperoside may work as a potential therapeutic agent or preventive remedy for Alzheimer's disease.

## Highlights

• HYP inhibits Aβ aggregation-induced neurotoxicity *via* direct binding to Aβ.
• HYP targets the endoplasmic reticulum-mitochondrial Ca^2+^ signal cascade through RyR2.
• Nasal administration increases bioavailability of HYP in APP/PS1 mice brain.
• HYP attenuates Aβ plaque deposition in the cortex and hippocampus of APP/PS1 mice.
• HYP improves learning and cognitive abilities in APP/PS1 mice.

## Graphical abstract

**Figure:**
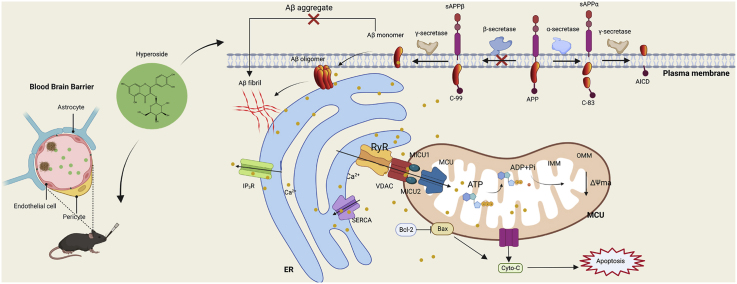

## Abbreviations

AD: Alzheimer's disease
Aβ: amyloid β-protein
APP: amyloid-beta precursor protein
HYP: Hyperoside
APP/PS1: APP/PSEN1
WT: Wild-Type
BACE: Beta-secretase
cyto-c: cytochrome c
Fig: Figure
ATP: adenosine triphosphate
ER: endoplasmic reticulum
ThT: Thioflavin T
ThS: Thioflavin S
BLI: Biolayer Interferometry
MRM: multiple reaction monitoring
mRNA: Messenger RNA
MCU: mitochondrial calcium uniporter
Q-PCR: Quantitative Polymerase Chain Reaction
MTT: 3-(4,5-dimethylthiazol-2-yl)-2,5-diphenyltetrazolium bromide
PI: Propidium Iodide
DMSO: Dimethyl sulfoxide
IS: internal standard
ISO: Isorhamnetin
BBB: blood-brain barrier
CNS: central nervous system
NMDAR: N-methyl-d-aspartate receptor
MEM: Memantine Hydrochloride
GFAP: glial fibrillary acidic protein
CA1: cornuammonis region 1
CA3: cornuammonis region 3
DG: dentate gyrus region
OMM: outer mitochondrial membrane
VDAC1: voltage-dependent anion channel 1
HFIP: hexafluoroisopropanol
SSA: super streptavidin
JC-1: 5,5′,6,6′-tetrachloro-1,1′,3,3′-tetraethylbenzimidazolyl cobalt iodide
CCCP: Carbonyl cyanide m-chlorophenyl hydrazine
EP tube: Eppendorf Micro Test Tubes

## 1 Introduction

Alzheimer's disease (AD) is the most common form of dementia, and the number of people with dementia is expected to reach 152 million by 2050 [1]. While most patients with AD have amnestic problems, a significant proportion of young-onset cases have atypical phenotypes including predominant visual, language, executive, behavioral or motor dysfunction [2,3]. Currently, scientists have proposed multiple hypothesis for the pathogenesis of AD, for example, the amyloid β (Aβ) hypothesis has suggested amyloid biomarkers as the earliest evidence of detectable neuropathological changes in AD patients [[4], [5], [6]]. With the continuous research and development on AD biomarker and its application in diagnostic setting, Jack and colleagues [7] proposed the “ATN” framework, which classified biomarkers into A (amyloid), T (phosphorylated tau) and N (neurodegeneration and neuronal injury) in 2018. In this research framework, the diagnosis of Alzheimer's disease is defined by the presence of phosphorylated tau and amyloid β. In fact, the earliest cellular pathogenesis of Alzheimer's disease included both the existence and accumulation of Aβ and p-tau [8]. Therefore, although the role of Aβ on AD remains controversial, diagnosis (by PET scans and plasma assays) on the level of Aβ and phosphorylated tau remains one of the most classical way for clinical and research work on AD [1]. Since Aβ may not be the only culprit in the pathology of AD, other emerging putative causes of AD ranging from inflammation to metabolic dysfunction, and also tau pathology have also been suggested as the possible pathogenic mechanisms for AD [9]. Tau, the microtubule-associated protein becomes hyperphosphorylated and aggregates into neurofibrillary tangles in AD patients' brains [10]. Insoluble tau aggregates are highly associated with the cognitive and clinical symptoms of AD [11]. Therefore, current pharmacological research and treatments are still poised at the advanced stages of clinical trials by targeting on anti-amyloid β, anti-tau, and anti-inflammatory strategies [12]. In current clinical diagnosis strategy, the presence of amyloid β (regardless of the presence of phosphorylated tau or neurodegeneration) which is cleaved from the larger precursor amyloid precursor protein (APP), remains one of the key markers for the diagnosis of AD pathological change [13]. Cleavage of APP by β-secretase (BACE) produces sAPPβ and C99. γ-secretase is also proposed to play a role in protein hydrolysis of Notch [14], which cleaves into the pathogenic Aβ (1–42) [15]. Aβ (1–42) spontaneously aggregates into β-sheet-rich oligomers and fibrils, which the Aβ oligomers are reported to be transient intermediates in the formation of protofibrils [15] and did not exist as stable entities [16]. With the uncertain identity and pathogenic mechanisms of Aβ aggregates in AD [17], recent studies showed a robust correlation between the soluble Aβ oligomer levels and severity of cognitive impairment [[18], [19], [20]]. There is also evidence that the accelerated Aβ fibrillation process greatly enhanced the toxicity of Aβ *in vitro* [21,22].

Furthermore, Aβ neurotoxicity has also been associated with intraneuronal Ca^2+^ dyshomeostasis [23]. The calcium hypothesis [24,25] of AD suggested that the activation of amyloid pathway remodels neuronal Ca^2+^ signaling, since Aβ aggregates can bind strongly to neuronal cell membranes [26,27]. Following an increase in cytoplasmic Ca^2+^, excess Ca^2+^ uptake into mitochondria leads to overload of Ca^2+^, inhibition of ATP synthesis, opening of the mitochondrial permeability transition pore (mPTP), release of cytochrome *c* and apoptosis [28]. Reduction of intracellular calcium significantly lowered the cytotoxicity level. In addition, it is also shown that a normal Ca^2+^ balance is responsible for the mechanisms of learning and memory [29]. As shown by quantitative imaging [30], 20% of neuronal Ca^2+^ was elevated (calcium overload) in APP/PSEN1 (APP/PS1) mice with cortical plaques, compared with less than 5% in wild-type mice. The ryanodine receptor (RyR) 2 protein is a major component of Ca^2+^ channels located in the sarcoplasmic reticulum, allowing calcium release from the sarcoplasmic reticulum into the cytoplasmic matrix [31]. RyR2 channels are implicated in many cellular functions, particularly mitochondrial metabolism, and disruption of RyR2 regulation in the endoplasmic reticulum (ER) mediated the key signal transduction cascade responses associated with AD [24]. Hyperoside (HYP), as the main component of *Crataegus pinnatifida Bunge,* is a folk medicine and multifunctional medicine food homology agent reported for its antidepressive, antitumor and antiviral effect [32], however, its neuroprotective effect remains unexplained. In this study, the protective role and mechanisms of HYP in reducing neuronal cell death through anti-Aβ and targeting ER-mitochondrial Ca^2+^ signaling cascade response were firstly reported. In addition, behavioral evaluation on APP/PS1 double transgenic AD mice model showed that HYP improved cognitive and learning functions, reduced Aβ plaques, and attenuated apoptosis in the cortex and hippocampus of mice via modulation of Ca^2+^ signaling cascade. In summary, this study has provided scientific evidence for development of HYP as a potential natural therapeutic agent for AD.

## 2 Materials and methods

### 2.1 Rengeats

All chemicals and reagents were purchased from Sigma unless otherwise stated. The following reagents from other suppliers were used: Hyperoside (PuFei De, Chengdu, CHINA), Aβ (1–42) (DGpeptites, Hangzhou, CHINA), BAPTA/AM (Santa Cruz, CA, USA), Thapsigargin (Sigma, MO, USA), Dantrolene sodium salt (Sigma, MO, USA), Thioflavin T (Sigma, MO, USA), Thioflavin S (Sigma, MO, USA), RIPA (CST, MA, USA), BACE1 Kit (Bioscience, CA, USA), FLIPR® Calcium 6 Evaluation Kit (Molecular Device, CA, USA). Apoptosis was detected by Annexin V staining kit (BD Biosciences, CA, USA). FlexiTube RyR2 siRNA target sequence: 5′-CTCGTCGTATTTCTCAGACAA-3′, Lipofectamine™ RNAiMAX Transfection Reagent (Thermofisher, CA, USA), Enhanced mitochondrial membrane potential assay kit with JC-1 (Beyotime, Shanghai, CHINA), Calcein AM Cell Viability Assay Kit (Beyotime, Shanghai, CHINA), Purified anti-β-Amyloid 1–16 Antibody (clone 6E10) (Biolegend, CA, USA), anti-amyloidogenic protein oligomer A11 (Invitrogen, CA, USA), anti-Amyloid Fibril antibody [mOC87] (Abcam, MA, USA), APP (E4H1U) Rabbit mAb against total APP protein (CST, MA, USA), BACE1 polyclonal antibody (Proteintech, IL, USA), purified anti-sAPPβ antibody (Biolegend, CA, USA) were used. Bax antibody (CST, MA, USA), Bcl-2 (D17C4) rabbit mAb (CST, MA, USA), cytochrome *c* antibody (A-8) (Santa Cruz, CA, USA), GFAP (D1F4Q) XP® Rabbit (CST, MA, USA), anti‐β‐actin mouse monoclonal IgG1 (Santa Cruz, USA), Agilent Zorbax Eclipse Plus C-18 column (Agilent, CA, USA) were also adopted.

### 2.2 Cell culture

Unless otherwise stated, all cells were obtained from the American Type Culture Collection (Thermofisher, CA, USA). All media were supplemented with 10% FBS and the antibiotics penicillin (50 U/mL) and streptomycin (50 μg/mL, Invitrogen, CA, USA). All cell cultures were incubated at 37 °C in a 5% humidified CO^2^ incubator.

### 2.3 Computational docking

Structure of HYP from Pubchem and Aβ (1–42) co-crystal structure from RCSB PDB were downloaded from the databases. In ligand preparation, Schrodinger Ligprep was used to prepare high quality ligand for further molecular docking. Protein was prepared using Schrodinger Protein Preparation Wizard for pre-processing, optimization, water removal and minimization procedures. Sitemap tools were used to evaluate potential protein binding sites. Receptor grid was generated using results of SiteMap in Receptor grid generation in the Glide application (Glide, version 9.1, Schrödinger) of Maestro (Maestro, version12.8, Schrödinger). The receptor grid for Aβ (1–42) was generated by specifying the binding (active) site residues, which was identified by SiteMap tool. Once the receptor grid is generated, the ligands are docked to the protein Aβ (1–42) using Glide version 9.1 (Grid based LIgand Docking with Energetics) docking protocol. Energy was calculated using the Calculate Energy module in MacroModel application (BatchMin V13.2).

### 2.4 Aβ peptide preparation

1 mg of Aβ (1–42) peptide was dissolved in 400 μL of hexafluoroisopropanol (HFIP; Sigma) and sonicated for 5 min. The Aβ peptide solution was aliquoted into 1.5 mL tubes (100 μL/tube) and nitrogen blown to dryness to produce a peptide film to yield Aβ monomer and stored at −80 °C. Aβ was re-dissolved in 10 μL of dimethyl sulfoxide (DMSO) (Sigma, USA) and an appropriate amount of PBS (pH = 7.4) to the desired final concentration before use. The Aβ peptides were incubated at 37 °C for 5 days to promote the aggregation to form Aβ aggregates.

### 2.5 Characterization of enriched Aβ aggregates

The intensity-weighted average hydrodynamic diameter of Aβ aggregates (30 μM) in PBS at 25 °C was measured with the Zetasizer Nano ZSP (Malvern Instruments) at a backscattering angle of 173° versus the polydispersity index PDI (a parameter used to describe the width of the size distribution) and the diameter number distribution curve [33].

### 2.6 Thioflavin T (ThT) fluorescence assay

HYP was incubated with Aβ (30 μM) for 7 days at 37 °C at a final volume of 100 μL. ThT fluorescence measurement was measured for every 24 h. ThT (20 μM) dissolved in PBS (pH = 7.4) was added in a black 96-well plate with 10 μL of aggregated Aβ (with or without HYP) and incubated for 1 h. Fluorescence measurement was then performed using a microplate reader (SpectraMax Paradigm, Molecular Devices, CA, USA) with an excitation wavelength of 450 nm and an emission wavelength of 490 nm. Background fluorescence was measured in control samples containing PBS and 0.02 % DMSO.

### 2.7 Biolayer interferometry (BLI) analysis

200 μL of solution containing 200 μg of the Aβ peptide was incubated at 37 °C for 5 days. EZ-Link NHS-LC-LC-Biotin (Thermo Scientific, USA) was dissolved in DMSO to a concentration of 10 mM. Aβ aggregates was biotinylated in a 1:0.5 M ratio of biotin reagent and incubated for 30 min at room temperature before being added to a 96-well plate (Greiner Bio-One, PN:655,209). Biotinylation was ascertained by loading the mixture onto super streptavidin (SSA) capacity tips (ForteìBIO, CA, USA) and detected by the FortéBIO Octet Red instrument. Additionally, SSA biosensors were pre-wetted with PBS for the recording of baselines. Successful biotinylated Aβ aggregates solution was collected and immobilized onto SSA tips overnight at 4 °C. HYP dissolved in DMSO was diluted to an appropriate concentration with PBS to a final 200 μL/well volume. Control wells were added with an equal amount of DMSO. All experiments consisted of repeated cycles of four significant steps: wash (300 s), baseline (120 s), association (120 s), and dissociation (120 s). The association, dissociation plot, and kinetic constants were analyzed with ForteìBIO data analysis software.

### 2.8 Cell membrane permeability assay

HT22 cells were co-treated with Aβ aggregates and HYP (20–80 μM) for 24 h and washed with PBS for 3 times. The cell membrane was disrupted with 200 μL methanol for the collection and centrifugation of cellular content at 20,000 rpm for 10 min. The supernatant was then collected for LC-MS/MS analysis.

### 2.9 BACE-1 inhibition assay

The assay was performed in a 96-well flat-bottom white plate using β-Secretase Activity Fluorometric Assay Kit (Biovision, CA, USA). To begin, HT22 cells were treated with HYP (20–80 μM) for 24 h and then collected by centrifugation with ice-cold extraction buffer added for homogenization. After centrifugation, 50 μL supernatant (cell lysate) was transferred to each well in the 96-well plate. 2 μL of active β-secretase (protein concentration: 4 μg/μL) was added to the 50 μL of extraction buffer as the positive control. For negative control, 2 μL of the β-secretase inhibitor was added to the 50 μL of sample well. 2 μL of each of the tested samples were added into each sample well for inhibitory activity evaluation. Following compounds addition, 50 μL of 2X reaction buffer was added with a gentle mix and incubation for 20 min at 37 °C before adding 2 μL of the β-secretase substrate. The plate was then covered and incubated in the dark at 37 °C for 1 h. Samples were then measured in a fluorescent 96-well plate reader (Ex/Em = 345/500 nm). Background readings produced from the substrate without adding secretase were subtracted from all samples.

### 2.10 Protein extraction and western blotting

After HYP treatment, the cells were lysed with RIPA. Protein concentrations were determined by Bio-Rad protein assay (Bio-Rad Laboratories, CA, USA). After electrophoretic separation, the gels were blotted and stained with primary antibodies. Binding of antibodies was visualized with peroxidase-coupled secondary antibodies using ECL Western Blotting detection reagents (Invitrogen, Scotland, UK). Band intensities were quantified using ImageJ software. Data were obtained from three independent experiments.

### 2.11 Dot blot assay

4 μL of prepared cell or animal sample protein solutions were dotted for adsorption onto methanol-pre-activated PVDF membranes and then blocked with 5% non-fat dry milk in Tris-buffered saline and Tween 20 for 1 h. The samples were incubated overnight at 4 °C with anti-amyloid fibril primary antibody [mOC87] (Abcam, MA, USA), anti-amyloid oligomer A11 (Invitrogen, CA, USA) or purified anti-β-amyloid (1–16) antibody (clone 6E10) (Biolegend, CA, USA) (1:1000), followed by HRP-conjugated secondary antibody. Protein bands were detected using Super Signal Sensitive ECL Western Blotting Detection Reagent (Beyotime, Beijing, China) and observed using a gel imaging device (Amersham Imager 800, GE, Tokyo, Japan).

### 2.12 Cytotoxicity assays

Cytotoxicity was assessed using the MTT (5.0 mg/mL) assay. 4 × 10^3^ HT22 cells per well were seeded in 96-well plates. After overnight incubation, cells were treated with Aβ aggregates (30 μM) for 48 h with or without the presence of HYP (20–80 μM). Cells were treated with DMSO as a control. Subsequently, MTT (10 μL) was added to each well for 4 h, followed by the addition of 100 μL of lysis buffer (10% SDS in 0.01 mol/L hydrochloric acid) and overnight incubation. The absorbance was measured at 570 nm the next day. The formula was calculated as a percentage of cell viability: Cell viability (%) = A (treatded) / A (control) × 100%. Data were obtained from three independent experiments in triplicate.

### 2.13 LIVE/DEAD cells analysis

According to the manufacturer's instructions, cell death was detected by Calcein AM Cell Viability Assay Kit (Beyotime, Shanghai, CHINA). 5 × 10^4^ HT22 cells per well were seeded in 24-well plates, and HT22 was treated with HYP (20–80 μM) or PBS for 24 h. After overnight incubation, treated cells were stained with Calcein/Propidium Iodide (PI) dye for 20 min. Cells images were then visualized and captured by using Olympus IX71 fluorescence microscope with FITC and TRITC filters consecutively. Green and red fluorescence images were merged and analyzed with cellSens Standard 1.8.1 software. The percentage of cell death was quantified by dividing the number of dead cells (red fluorescence) by the total number of cells.

### 2.14 Annexin V detection by flow cytometry analysis

Apoptosis was detected with Annexin V staining kit (BD Biosciences, CA, USA). Briefly, HT22 cells were treated with HYP (20–80 μM) and Aβ aggregates (30 μM) for 24 h were stained with FITC-Annexin V and PI for flow cytometric detection according to the manufacturer's instructions. The number of apoptotic cells was quantified by flow cytometry (BD FACSAria III, CA, USA). Data acquisition and analysis were performed with CellQuest (BD Biosciences) from three independent experiments.

### 2.15 Fluorescent probe method (Fluo-3) to detect calcium ion concentration

2 × 10^5^ HT22 cells were incubated in 35 mm confocal dishes for 24 h at 37 °C in a CO^2^ incubator. A 5 mM Fluo-3 AM stock solution was diluted with Hanks-Balanced Salt Solution (HBSS) to a 5 μM working solution and then added to the cells for 30 min at 37 °C. HT22 cells were washed three times with HEPES-buffered saline and then incubated in an imaging chamber at 37 °C for an additional 10 min. After the addition of 30 μM of Aβ aggregates in HBSS buffer, changes in cellular Ca^2+^ levels were monitored by epifluorescence microscopy (Applied Precision DeltaVision Elite, Applied Precision, Inc., United States) using the real-time mode for tracking Fluo-3 changes for 5 min. The data inspection program provided by DeltaVision software was used to measure the intensity of Fluo-3 fluorescence.

### 2.16 Measurement of cytoplasmic calcium dynamics

Intracellular Ca^2+^ dynamics were determined using the FLIPR Calcium 6 Assay Kit (Molecular Devices) according to the manufacturer's instructions. Briefly, HT22 was housed at 4 × 10^4^ cells per well in a black-walled and clear-bottom 96 multi-well plate (Costar, MA, USA) and treated with Calcium 6 reagent for 1 h. A mixture of HYP and Aβ aggregates or thapsigargin (TG) was added to the wells and data were immediately collected at room temperature using SpectraMax Paradigm multi-mode microplate reader (Molecular Devices, CA, USA) using 5-s read intervals in five independent experiments.

### 2.17 siRNA transfection

HT22 were transfected with siRNAs using Lipofectamine™ RNAiMAX Transfection Reagent (Thermofisher, CA, USA) according to the manufacturer's protocol. To maximize the knockdown efficiency for the RyR2, the siRNA of different RyR2 isoforms were used for transfection. RyR2 siRNA target sequence 5′-CTCGTCGTATTTCTCAGACAA-3′ was purchased from Qiagen (CA, USA).

### 2.18 Real‐time quantitative PCR

RNA was extracted from the HT22 cell using FavorPrep™ Blood/Cultured Cell Total RNA Purification Mini Kit (Favorgen Biotech Corp.). RNA concentration was determined using the NanoDrop 2000c Spectrophotometer (Thermo Scientific). 1 μg of total RNA was used to reverse transcribe to its corresponding cDNA by using the Transcriptor Universal cDNA Master mix (Roche, USA). Quantitative PCR was then performed with the addition of PowerUp™ SYBR® Green Master Mix (Applied Biosystems) using the ViiA™ 7 Real-Time PCR System (Applied Biosystems). Specific primers (Tech Dragon Ltd., Hong Kong) were designed by employing ThermoFisher Scientific's online OligoPerfect™ Designer software and then verified with NCBI's Primer‐BLAST software to confirm specific recognition of the target genes. Gene expression levels were normalized to actin (control) and analyzed using the ΔΔCT method. Three independent experiments with three replicates per group were analyzed for each primer. Primer sequences are specified as in Table 1:

**Table 1:** Primer sequences.

Gene Name	Forward	Reverse
RyR2	5′-AGAAGGAGAGGCCAGAGGAG-3′	5′-GGACAGGGTTGGTCATGAGG-3′
APP	5′-CCTCCGTGTGATCTACGAGC-3′	5′-GAACCTGGTCGAGTGGTCAG-3′
MCU	5′-ACGACAACTGCAAGAGGAGG-3′	5′-CAGGGTCTTCACGTCGTTCA-3′

### 2.19 Measurement of mitochondrial membrane potential

5 × 10^4^ HT22 cells per well in 24-well plates were co-treated with Aβ aggregates and HYP (20–80 μM) or PBS for 24 h 10 mM of Carbonyl cyanide m-chlorophenyl hydrazine (CCCP) was used as a positive control. The mitochondrial membrane potential of HT22 stained with 5,5′,6,6′-tetrachloro-1,1′,3,3′-tetraethylbenzimidazolyl cobalt iodide (JC-1) was observed by laser scanning confocal microscopy.

### 2.20 ATP content measurement

HT22 cells were co-treated with Aβ aggregates and HYP (20–80 μM) for 24 h and washed with PBS. Each digested sample was re-suspended in 10 mL of ice-cold PBS, followed by centrifugation for 5 min (300 g, 4 °C). Cell pellets were then treated with 85% cold methanol (2 × 10^6^ cells / 100 μL) containing 2 μM ATP-^13^C10,^15^N5, then vortexed for 1 min and placed on ice for 10 min. After centrifugation (13,000 g) at 4 °C for 15 min, the supernatant was transferred into another tube. The derivatization reaction was initiated by adding 75 μL of derivatization reagent MTBSTFA into 200 μL of cell lysis with 85% methanol and completed over 5 min with consistent vortex. Derivatization samples were then centrifuged (13,000 g) at 4 °C for 10 min, and 25 μL of the supernatant was injected into LC-MS/MS system for analysis.

### 2.21 Pharmacokinetics analysis

After tail vein injection or nasal administration, blood was collected at 5, 15 and 30 min, 1, 2, 4, 8, 12, 24, 48, 72, 120 and 168 h, for immediate centrifugation at 14,000 rpm for 5 min. The serum was harvested and stored at −20 °C until further processing. Brain tissue was collected and was thoroughly rinsed in saline to eliminate blood and blotted dry with filter paper. Each tissue sample was homogenized in saline (1:5, w/v) and stored at −20 °C until analysis.

### 2.22 Determination of serum and brain tissue drug concentrations

50 μL of serum or 100 μL of brain tissue was put in a 1.5 mL EP tube, followed by the addition of 20 μL of internal standard solution (10 μM ISO). The protein was precipitated with 1 mL of ethyl acetate, vortexed for 5 min and centrifuged at 20,000 g (4 °C and 10 min). The supernatant solution was nitrogen blown to dry and re-suspended with 100 μL of 50% acetonitrile for centrifugation again. The supernatant was taken for analysis by using LC-MS/MS.

### 2.23 LC-MS/MS measurement of HYP

HYP content was quantified using UHPLC-MS/MS system, which includes Agilent 1290 Infinity UHPLC, and Agilent 6460 Triple Quadrupole, equipped with an electrospray ionization interface used to generate positive ions for the determination of HYP. The compounds were analyzed by using the Agilent Zorbax Eclipse Plus C-18 column with a particle size of 1.8 μM (flow rate: 0.35 mL/min). The mobile phase was set as follows: mobile phase A (0.1% formic acid in water) and mobile phase B (0.1% formic acid in ACN): 0–3 min, 10–30% B; 3–4 min, 70% B; 4–5 min, 30–70% B; 5–6 min, 70-10% B. The column and auto-sampler temperature were maintained at 30 °C and 4 °C, respectively. Data were analyzed by using Agilent MassHunter Workstation software B.01.03. The gas temperature was set at 300 °C with a flow rate of 11 L/min. Gases were set at 30 psi for the nebulizer, capillary, at 4000 V. For the HYP, the fragment was 170. The collision energy was set at 30; The mass transitions were as follows based on multiple reaction monitoring: *m*/*z* 464.38 → 299.9. For the Isorhamnetin (ISO), the fragment was 150. The collision energy was set at 30; The mass transitions were as follows based on multiple reaction monitoring: *m*/*z* 315.4 → 300.1, The measurements of HYP were done using the standard and linear least-squares regression curve.

### 2.24 Animal experiment

All animal care and experimental procedures were performed in accordance with the “Institutional Animal Care and User Committee Guidelines” of the Macau University of Science and Technology. APP/PSEN1 (APP/PS1) mice (N000175) were purchased from the Nanjing Institute of Biomedical Sciences, Nanjing University, China, weighing 25 ± 5 g. The animals were housed in a temperature-controlled room with a 12 h light/dark cycle and given ad libitum food and water. Male mice were randomly divided into the following 6 experimental groups. (a) wild-type group (n = 6), (b) APP/PS1 group (n = 6), (c) HYP (20 mg/kg group) (n = 7), (d) HYP (40 mg/kg) group (n = 8), (e) HYP (80 mg/kg) group (n = 8) and (f) memantine (MEM) (10 mg/kg) positive control group (n = 8). HYP was dissolved in the solvent containing 10% DMSO, 40% PEG300 and 5% Tween-80 in PBS, and administrated via nasal injection for every 3 days with a volume of 0.5 μL/g of body weight for 8 consecutive weeks.

### 2.25 Y-maze tests

Y-maze tests assess cognitive changes, short-term spatial working memory (by spontaneous alternation), and exploratory activity (by total number of arm choices) of mice. The Y-maze apparatus (Yuyan, Shanghai, China) is a three-arm horizontal maze (40 cm long, 10 cm wide with 12 cm high walls) in which the arms are symmetrically disposed at 120° angles from each other. To begin, mice were placed at the end of one arm and allowed to move freely through the maze during a 9-min session. The number of total arm choices and sequence of arm choices were recorded. The percentage of alternation can be calculated as the ratio of total number of alternations / the number of arms entered x 100%. Before each trial, the interior of the maze was sprayed with 70% ethanol solution to erase any scent cues [34].

### 2.26 Fear-conditioning tests

The fear conditioning test measures the ability of mice to learn and remember associations between aversive experiences and the environment. On the first day of the fear conditioning test (Yuyan, Shanghai, China), mice were placed in a chamber for 5 min to acclimatize and explore. On the second day, the training (fear conditioning phase) was performed by placing the mice into a standard operating chamber with sound attenuation for 3 min. A 30-s tone (3 kHz, 85 dB) was then delivered, followed by a 2-s electric shock (0.5 mA). The training was repeated twice over a 4-min period. 24 h later, the mice were placed in the same conditioning chamber for a context retention test which the conditions were consistent with the training except that the 2-s electric shock was not allowed. Autonomic activity was performed for 90 s after the tone stimulus, for a total of 5 min. Responses were recorded with a video camera and scored for resting time, which was defined as the absence of any movement other than breathing [35,36].

### 2.27 Rotarod test

The rotarod test was used to monitor motor coordination. The test consisted of placing the animal on a rotating rod with a rotational speed of 25 rpm (Yuyan, Shanghai, China) until the animal fell to the ground and the movement time was recorded. The test was performed three times with an interval of 300 s, and the mean of the test results were calculated.

### 2.28 Hematoxylin and eosin (HE) staining

Pathological assessment on the cornuammonis region 1 (CA1), cornuammonis region 3 (CA3) and dentate gyrus region (DG) regions of the mouse hippocampus was done by HE staining. Brain sections were dehydrated with 70%, 80% and 90% alcohol respectively. Sections were then subjected to staining with hematoxylin (50 °C) for 30 s, incubation with 1% hydrochloric acid alcohol for 10–20 s, washing with 0.5% ammonia hydroxide for 10 s, staining with eosin for 3–5 s, and finally dehydration in 70%, 80%, and 90% alcohol respectively. After transparentized within dimethyl benzene, the sections were sealed with neutral gum for microscopic observation. Images were captured by light microscopy (Leica, WZ, GER).

### 2.29 Nissl staining

The CA1, CA3 and DG regions of the mouse hippocampus was assessed by Nissl staining for Nissl body detection. Brain sections were dehydrated as the same as in HE staining, followed by 1% tar violet staining for 1 h, and with distilled water washing followed by 70% alcohol separation for 1 min. Tissues were then dehydrated in 70%, 80% and 90% of alcohol. After transillumination in dimethylbenzene, sections were sealed with neutral adhesive for microscopic observation. Images were captured by a light microscope (Leica, WZ, GER).

### 2.30 Thioflavin S (THS) staining

Paraffin sections of the brain were washed three times with PBS after dewaxing and then stained with THS dissolved in 50% alcohol for 7 min, followed by 50% alcohol solution for washing. Sections were then air-dried and sealed with neutral gel for microscopic observation. Images were captured by API DeltaVision Live‐cell Imaging System (Applied Precision Inc., DC, USA).

### 2.31 Immunofluorescent staining of brain tissues

Brain tissues from all treatment groups were fixed and embedded in paraffin for microtome sectioning and immunofluorescence staining. After deparaffinization, the tissue sections were subjected to antigen retrieval (EnVision™ FLEX Target Retrieval Solution, High pH), soaked in PBS, dehydrated, deparaffinized and rehydrated according to the standard protocols. Next, the sections were incubated in 1% Triton X-100 for 40 min, rinsed with PBS, and blocked with 5% bovine serum albumin (BSA) for 40–60 min. The sections were incubated with anti-GFAP rabbit polyclonal antibodies (1:200, CST, CA, USA) at 4 °C overnight. After rinsed with PBS, the sections were incubated with fluorescein secondary antibodies (1:200, CST, CA, USA) for 1 h, then counterstained with DAPI for 10 min. The coverslips were then mounted with FluorSave™ mounting media (Calbiochem, San Diego, CA, USA) for fluorescence imaging. The expression of GFAP was captured by API DeltaVision Live‐cell Imaging System (Applied Precision Inc., GE, DC, USA).

### 2.32 Microarray data analysis

An Agilent Microarray Scanner (Agilent Technologies) was used in the present study. Data were obtained using Feature Extraction software 10.7 (Agilent Technologies). Raw data were normalized by Quantile algorithm, Gene Spring Software 11.0 (Agilent Technologies). The mRNAs were considered to be differentially expressed when the fold change was >2 (p < 0.05). A volcano plot was used to visualize differentially expressed genes and was subsequently processed for hierarchical clustering analysis using Gene Spring Software 11.0 (Agilent Technologies). Finally, Pearson correlation coefficients between differentially expressed mRNAs were calculated, and co-expression networks of mRNAs were constructed.

### 2.33 Gene ontology (GO) and kyoto encyclopedia of genes and genomes (KEGG) pathway analysis

GO terms were used to annotate and classify gene function. The differentially expressed genes were put into the Database for Annotation, Visualization and Integrated Discovery (DAVID; http://david.abcc.ncifcrf.gov/) v6.8, which utilizes GO to identify the molecular function represented in the gene profile. Furthermore, KEGG was used to analyze the potential functions of these genes in metabolic pathways. p < 0.05 was recommended as a cut-off value.

### 2.34 GSEA analysis of differentially expressed genes

The GSEA analysis was done using GSEA software version 2.2.2.0 (49, 70), which used predefined gene sets from the Molecular Signatures Database (MSigDB v5.0) (70). A gene set is a group of genes that shares pathways, functions, chromosomal localization, or other features. For the present study, the C collection sets for GSEA analysis (i.e., C1–C7 collection in MsigDB) and list of ranked genes based on a score calculated as −log10 of P value multiplied by sign of fold-change were used. The minimum and maximum criteria for selection of gene sets from the collection were 10 and 500 genes, respectively.

## 3 Results

### 3.1 Verification of the binding propensity of HYP to Aβ

**Fig. 1:**
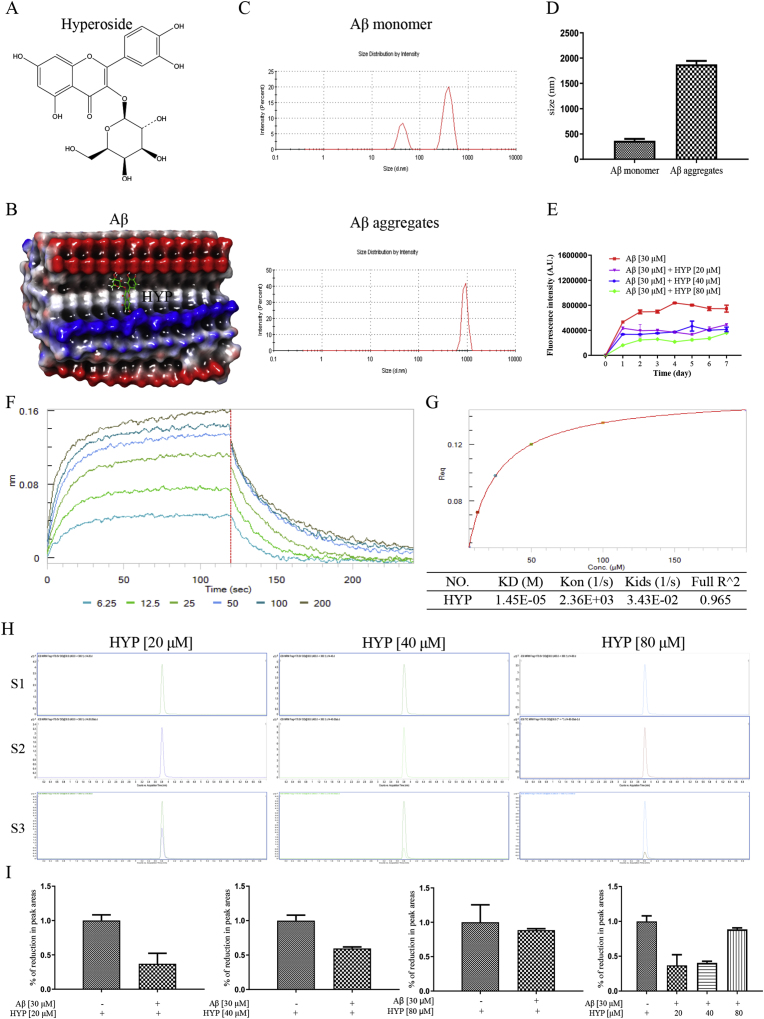
The binding propensity of HYP to Aβ (**A**) Chemical structure of HYP. (**B**) Computational docking of HYP with Aβ (2MXU). HYP and Aβ (2MXU) were represented as green sticks and red ribbons, respectively. (**C**) ZetaView measurements of the mean hydrodynamic diameter of Aβ monomer and Aβ aggregates. (**D**) Particle size analysis of Aβ monomer and Aβ aggregates. (**E**). Measurement of Aβ (1–42) fibrillation. Fluorescence intensity (A.U.) of fibrillated Aβ (1–42) from the indicated treatment groups were monitored from day 1–7 by ThT assay. (**F**) Kinetic binding sensorgrams of increasing concentrations of HYP from 6.25 to 200 μM. The Y axis (nm) represented the optical thickness changes of the Aβ sensor layer during the interaction of HYP and Aβ. (**G**) Steady-state analysis of the binding curves and measured parameters of the binding interaction: binding rate constant (Kon), dissociation rate constant (Kids), and affinity constant (KD). (**H**) MRM chromatograms of HYP-treated HT22 cells. S1: HYP (20, 40 or 80 μM) alone groups. S2: 30 μM aggregated Aβ co-incubated with 20, 40 or 80 μM of HYP groups; S3: Overlaid chromatograms of S1 and S2. (**I**) The quantitative reduction (%) of HYP peak area in MRM chromatograms of HYP (20–80 μM) with or without the co-incubation of 30 μM aggregated Aβ. All data were mean ± S.D. compared with the HYP alone groups, n = 3. *P < 0.05, **P ≤ 0.01, ***P ≤ 0.001, one-way ANOVA analysis. (For interpretation of the references to colour in this figure legend, the reader is referred to the Web version of this article.)

Quercetin-3-O-β-d-galactopyranoside (Hyperoside, HYP), belongs to the class of flavonol glycosides and is derived from *Crataegus pinnatifida Bunge* [37] (a hawthorn in the Rosaceae family), was shown in Fig. 1A. Aβ is a well-known biomarker correlated to AD, therefore, the effect of HYP in targeting Aβ was investigated by molecular docking. As shown in Fig. 1B, a binding energy of −8.9 kcal/mol between Aβ fibers and HYP was predicted by BatchMin V13.2. HYP with Aβ molecular surface interaction and hydrogen bonding information were shown in supplementary 1A and 1B. The ligand interaction diagram indicated that the hydroxyl group of HYP bind to Aβ by chain E-F (supplementary 1C). The formation of Aβ aggregate and its particle size was verified by DLS (Fig. 1C). While the average particle size of the Aβ monomer is 365.62 nm, the particle size of Aβ aggregate is 1877 nm (Fig. 1D). The detection on the formation of Aβ aggregate was then performed by using the ThT fluorescence assay. ThT is a benzothiazole-like small molecule that specifically binds to amyloid fibrils [38]. As shown in Fig. 1E, HYP reduced Aβ fibrillation and aggregation. The direct binding affinity of HYP to Aβ aggregate was confirmed by the BLI assay. By applying an increasing concentration of HYP (6.25–200 μM), a dose-dependent direct association of HYP to the biotinylated Aβ aggregate was shown by the association / dissociation binding curve of HYP towards Aβ aggregate (Fig. 1F). Together with Fig. 1G presenting the steady-state analysis of the binding curves and the binding affinity (KD), association rate constant (Kon) and dissociation rate constant (Kids) of HYP to Aβ, the results showed that the KD value of HYP binding to Aβ aggregates was 14.5 μM. To further confirm the Aβ binding propensity of HYP, increasing concentration of HYP was incubated with 30 μM of Aβ aggregate. By using HYP alone (without Aβ aggregate incubation) as the control, all incubation mixtures were analyzed by using LC/MS/MS under the same chromatographic conditions according to our previous reported detection method [39]. The quantitative reduction (%) in the peak area, representing the Aβ binding propensity of the tested compound [39] is used for statistical analysis. Fig. 1H showed the MRM of HYP alone (S1), HYP (20–80 μM) with the incubation of Aβ aggregates (S2), and the merged image (S3), with the peak area of all the identified components calculated and analyzed. As the concentration of HYP increased, Fig. 1I showed the percentage (%) of decrease in the peak area of HYP in MRM increased, indicating the dose-dependent binding affinity of HYP to Aβ aggregate. These data confirmed the Aβ binding propensity of HYP by both spectroscopic and chemical approaches.

### 3.2 HYP reduces BACE1 enzymatic activity and the level of both APP-β and Aβ

**Fig. 2:**
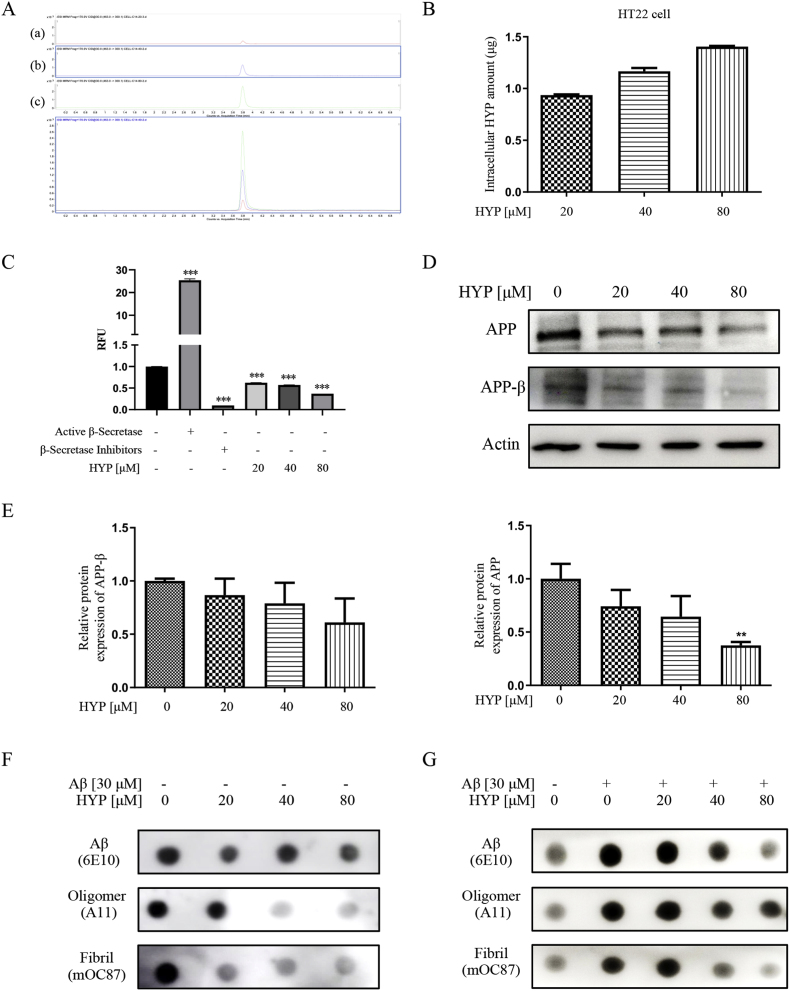
Inhibitory effect of HYP on Aβ level and aggregation (**A**) MRM chromatograms of HT22 cells after 24 h of different concentrations (a:20 μM, b:40 μM, c: 80 μM) of HYP treatment, and (**B**) the quantification of HYP peak area in MRM chromatograms. (**C**) mRNA level of APP in HYP treated HT22 cells. Data were normalized to the expression level of β-actin in each corresponding sample. (**D**) Enzymatic activity of β-secretase in each treatment condition was presented as the relative fluorescence units (RFU) obtained after normalization to untreated cells. Cells were treated for 24 h as the conditions indicated. (**E**–**F**) The protein expression and quantitative analysis of APP and APP-β level of HYP treated HT22 cells. (**G-H**) Measurement of the level of Aβ (Aβ [[1], [2], [3], [4], [5], [6], [7], [8], [9], [10], [11], [12], [13], [14], [15], [16]], Aβ oligomers and Aβ fibrils) in HYP treated HT22 cells with or without the addition aggregated Aβ. All data were expressed as mean ± S.D. compared to untreated control group, with n = 3. *P < 0.05, **P ≤ 0.01, ***P ≤ 0.001, one-way ANOVA analysis.

The intracellular level of HYP was measured by LC-MS/MS (Fig. 2A) to evaluate the penetration rate of HYP into HT22 cells. As shown in Fig. 2B, the intracellular level of HYP was increased upon the escalating concentration of HYP incubated with HT22 cells. In the early pathogenesis of AD, enzymatic cleavage of APP by BACE1 followed by γ-secretase contributed to the formation of pathogenic Aβ peptides. To investigate the effect of HYP in altering the level of Aβ, the mRNA level of APP in HT22 was evaluated after HYP treatment. While HYP has no significant effect on the mRNA transcriptional level of APP (Supplementary 1D), it inhibited β-secretase activity in HT22 cells (Fig. 2C), indicating a potential effect on reducing the production of Aβ peptide via inhibition of BACE 1, which is responsible for cleaving APP to produce toxic amyloidogenic Aβ peptides [40]. Protein immunoblots showed that HYP reduced the protein level of both APP and APP-β (Fig. 2D–E). Furthermore, dot blot analysis was performed to detect the level of different form of Aβ in HT22 cells. As shown in Fig. 2F, HYP lowered the endogenous level of Aβ Oligomers (A11), Aβ Fibrils (mOC87) and total Aβ (1–16) (6E10) in cells. On the other hand, HYP also lowered the level of different form of Aβ in HT22 cells incubated with exogenous Aβ for 24 h (Fig. 2G). These experimental results suggested that HYP partially inhibited the amyloidogenic pathway via attenuating the activity of BACE1 enzyme, and resulting in a lower level of amyloidogenic Aβ in different forms.

### 3.3 Pharmacokinetics study on HYP

**Fig. 3:**
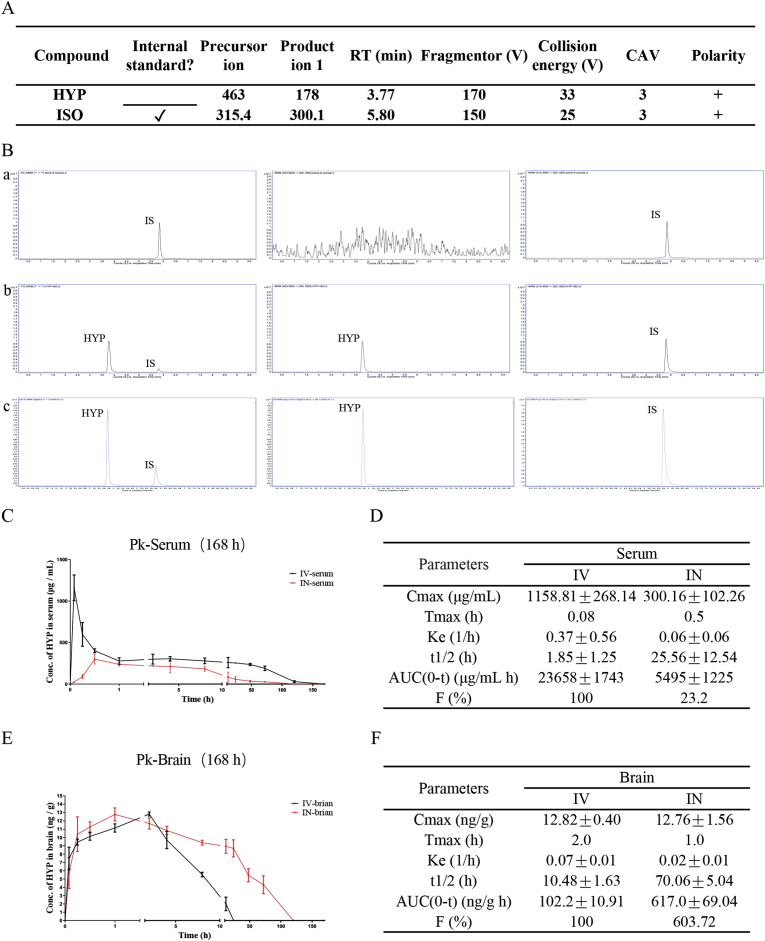
Representative chromatograms and pharmacokinetic parameters of HYP (**A**) MS/MS parameters for internal standard (ISO) and HYP. (**B**) The TIC chromatograms, MRM chromatograms of blank brain sample (a), HYP standard (b), and brain sample (c) were detected in homogenized mice brain samples, respectively. (**C**) The serum concentration-time profile of HYP after nasal administration or intravenous injections of 20 mg/kg in mice. (**D**) The main serum pharmacokinetic parameters of HYP in mice after nasal administration or intravenous injections. (**E**) The brain concentration-time profile of HYP after nasal administration or intravenous injections of 20 mg/kg in mice. (**F**) The main brain pharmacokinetic parameters of HYP in mice after nasal administration or intravenous injections. All data were expressed as mean ± S.D. (male, n = 5).

Before further study on the pharmacological efficacy of HYP *in vivo*, systematic pharmacokinetics study of HYP on mice was performed. To begin, Fig. 3A showed the LC-MS/MS information for HYP analyses and internal standards (IS). While the retention time for HYP was 3.77 min in both brain and serum samples, the retention time for ISO (IS) was 5.80 min. All detections were carried out using the predominantly positive ionization mode. Following detailed optimization of mass spectrometry conditions, *m*/*z* 463 → 178.4 was used for quantification of HYP and *m*/*z* 315.4 → 300.1 for IS. As shown in Fig. 3B, both HYP and ISO were well separated with sharp peaks. The TIC chromatograms, MRM chromatograms of control brain sample, HYP standard, and brain sample after HYP treatment were also obtained. The blood-brain barrier (BBB) strictly controls the transport of various substances from the blood to the brain [41], therefore many drugs with good therapeutic efficacy are ineffective in treating brain disorders due to its low BBB permeability and low therapeutic concentration that can reach the target brain region [42]. With the relatively low invasiveness and easily operation, nasal administration bypasses the BBB, increases the intracerebral bioavailability of the drug and avoids the first pass effect in liver and rapid absorption [43], therefore, nasal delivery method was adopted in this study for HYP delivery in mice.

HYP is a flavonol glycoside which is insoluble in water. In order to increase its solubility, HYP was dissolved in PBS (10% DMSO + 40% PEG300 + 5% Tween-80). The evaluation on the serum concentration of HYP in mice after intranasal (IN) or intravenous (IV) administration of 20 mg/kg was performed. The concentration-time profile of HYP in serum (Fig. 3C) indicated that the C_max_ value is 1158.81 ± 268.14 μg/mL; T_max_ is 0.08 h; t_1/2_ is 1.85 ± 1.25 h in serum after IV administration. On the other hand, the C_max_ value is 300.16 ± 102.26 μg/mL; T_max_ is 0.5 h; t_1/2_ is 25.56 ± 12.54 h in serum after IN administration. The AUC_IN_/AUC_IV_ ratio 23.2% for IN injection (Fig. 3D). These results suggested that after IN administration, the rate and degree of absorption of HYP in blood became lower when compared to IN administration, and the half-life was also significantly prolonged with a slower rate of elimination after IN injection. Of noted, with the IV administration bioavailability set as 100% [44], the bioavailability of IN administration in serum was confirmed as 23.2%. The brain concentration-time profile of HYP after IN or IV administration of 20 mg/kg in mice was also performed. As shown in Fig. 3E, the C_max_ value is 12.82 ± 0.40 ng/mL; T_max_ is 2 h; t_1/2_ is 10.48 ± 1.63 h in the brain after IV administration. In contrast, the C_max_ value is 12.76 ± 1.56 ng/m; T_max_ is 1 h; t_1/2_ is 70.06 ± 5.04 h in the brain after IN administration of HYP. The AUC_IN_/AUC_IV_ ratio is 603.72% after IN injection (Fig. 3F), suggesting a higher absorption rate of HYP in the brain tissue via IN injection. Although there is no significant difference in the degree of absorption as reflected by the Cmax value, the half-life (t_1/2_) was significantly prolonged. Besides, the elimination rate (Ke) of HYP was slower in IN administration, confirming the higher bioavailability of HYP in the brain via IN administration. By summarizing the above data, the rapid and favorable BBB permeability nature of HYP via IN administration were confirmed. Furthermore, based on the half-life value of HYP, a dosing interval of once for every 3 days was used in all the subsequent animal experiment via IN injection.

### 3.4 HYP improved spatial working memory and hippocampal memory in APP/PS1 mouse model

**Fig. 4:**
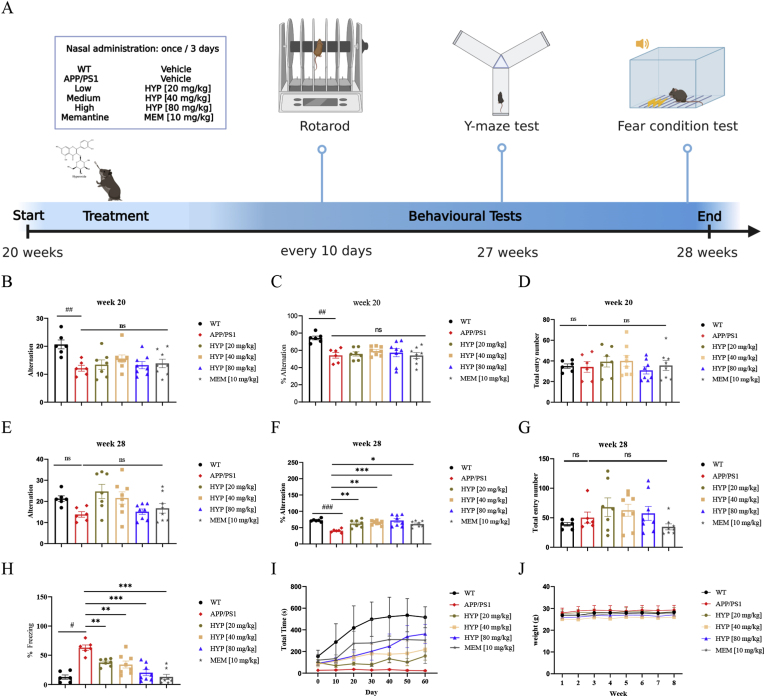
Effect of HYP in spatial working memory and hippocampal memory declines in APP/PS1 mouse model (**A**–**E**) Schedule of behavioral tests of APP/PS1 mice. (**A**) Schedule of behavioral tests and treatment conditions. 20 weeks old male APP/PS1 mice were nasal administrated with HYP (20 or 40 or 80 mg/kg, n = 7–8) or MEM (10 mg/kg, n = 8) or vehicle (10% DMSO+ 40% PEG300 + 5% Tween-80 in PBS, n = 6) for 8 consecutive weeks (once for every 3 days). Age-matched male WT (n = 6) mice were injected with vehicle as controls. (**B**–**D**) Y-maze tests on 20-week-old APP/PS1 mice before the experiment. The average alternation (**B**), percentage of alternation (%) for each tested group (**C**) and total entry number into each arm (**D**) on Y-maze. (**E**–**G)** Y-maze tests on 28-week-old APP/PS1 mice after nasal administration of HYP for 8 consecutive weeks. The average alternation (**E**), average percentage of alternation (%) for each tested group (**F**) and total entry number into each arm (**G**) on Y-maze were recorded. (**H**) Fear conditioning test was conducted with the fear response plotted as the % of freezing during the conditional stimulus. (**I**) Rotarod tests on both WT and APP/PS1 mice (n = 6–8). (**J**) Body weight of mice in all treatment groups. All data were mean ± S.D, n = 6–8, ^#^P < 0.05, ^##^P ≤ 0.01, ^###^P ≤ 0.001, compared with WT group; *P < 0.05, **P ≤ 0.01, ***P ≤ 0.001, compared with APP/PS1 group; one-way ANOVA analysis.

The most obvious feature of AD is the progressive decline in cognitive function caused by the progressive loss of neurons and synapses. Currently approved AD drugs including acetylcholinesterase inhibitors (donepezil) and N-methyl-d-aspartate receptor (NMDAR) antagonists (memantine hydrochloride) prescribed for relieving symptoms, however, are not able to improve the situation of neuronal cell death [45,46]. Literature has demonstrated that an overload of calcium was triggered by Aβ plaques in the APP/PS1 transgenic mouse model [30]. To confirm the neuroprotective effect of HYP *in vivo*, HYP was administered intranasally for 8 consecutive weeks to 20-week-old male APP/PS1 mice. In brief, 3 concentrations of HYP (20/40/80 mg/kg, n = 7–8) or MEM (10 mg/kg, n = 8) or vehicle (10% DMSO + 40% PEG300 + 5% Tween-80 in PBS, n = 6) were administrated. Age-matched male Wild-Type (WT) mice were also injected with vehicle (n = 6) for 8 weeks (Fig. 4A) as control. To assess the cognitive changes in APP/PS1 mice, the Y-maze spontaneous alternation test was performed at week 20 (Fig. 4B–D) and week 27 (Fig. 4E–G) at the beginning of the experiment to assess the initial learning and short-term spatial working memory before HYP treatment. In the Y-maze, mice were free to access the three arms in any order, and the number of consecutive accesses to the three different arms was measured and compared to the total number of accesses. The resulting alternation rate is considered as a measure of spatial working memory [47,48]. The total entry numbers and the number of alternations were calculated to assess motor capacity, and the percentage of alternations was used to evaluate the spatial working memory capacity. Fig. 4C showed that at week 20, the alternation rate was significantly lower in the APP/PS1 group when compared to the WT group, while locomotion was not affected (Fig. 4B–D), indicating that AD-like cognitive behavioral abnormalities occurred in these 20-week-old APP/PS1 mice. After 7 weeks of HYP administration, the spatial working memory of APP/PS1 mice was resumed to a level comparable to WT mice group (Fig. 4F). Furthermore, no observable behavioral differences were found in the HYP and vehicle-administered WT groups. Furthermore, possible confounding factors of cognitive improvement due to differences in behavioral abilities (Fig. 4E–G) were also excluded.

Next, emotion-related learning abilities based on the communication of amygdala-hippocampus, the main brain structure in the fear circuit involved in emotional memory formation and storage, and the hippocampus-dependent learning of fear-triggering context were assessed by measuring freezing responses in a fear conditioning test [36,49]. In Fig. 4H, while the freezing time was significantly increased in the APP/PS1 group, the HYP treatment significantly decreased the freezing time of APP/PS1 mice in the fear conditioning test, suggesting the improvement on the learning and memory ability. Finally, motor ability of the mice was examined by the rotarod test. As reflected by the total length of time that the mice retained on the rotating rod, Fig. 4I showed that the motor ability depending on the balance and coordination, as well as the physical condition of APP/PS1 mice was improved after HYP treatment. Throughout the *in vivo* experiment, all mice survived without significant changes in body weight (Fig. 4J) and organ index (supplementary 1E). *In vivo* behavioral data confirmed that the learning and spatial working memory, as well as motor ability of APP/PS1 mice were improved after HYP treatment.

### 3.5 HYP reduced Aβ plaques and aggregates in the cortex and hippocampus of APP/PS1 mice

**Fig. 5:**
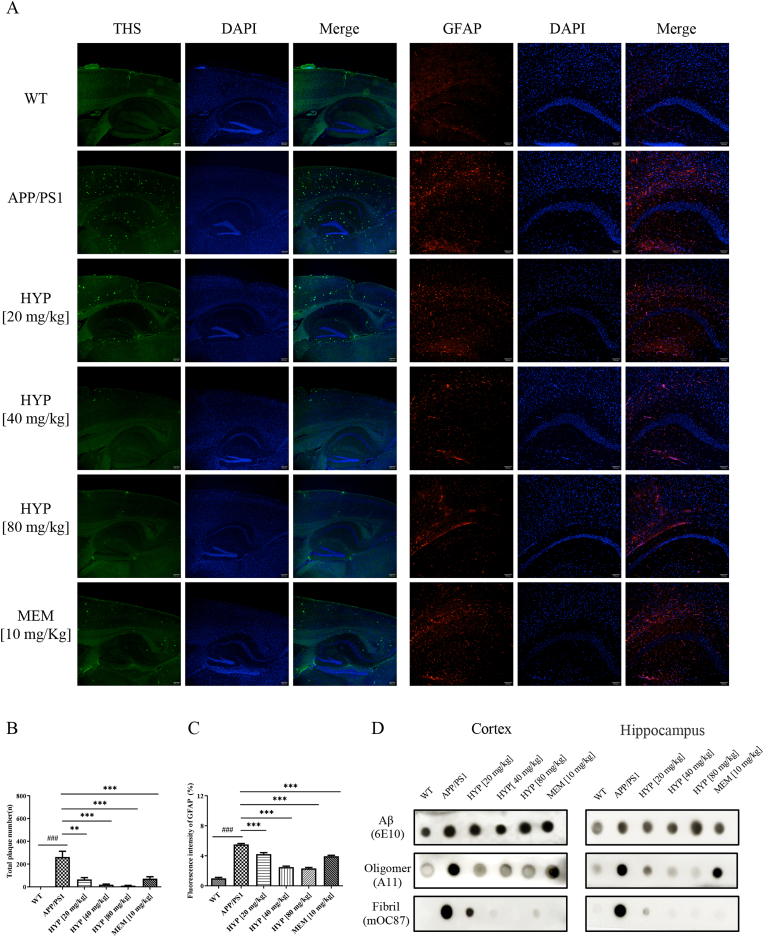
Pharmacological effect of HYP in the level of Aβ plaques and aggregates formation in the brains of APP/PS1 mice 28-weeks-old APP/PS1 (n = 6, male) and age-matched wild-type (WT) (n = 6, male) mice were nasal administrated with HYP (20 or 40 or 80 mg/kg, n = 7–8) or MEM (10 mg/kg, n = 8) at a frequency of once for every three days in 8 consecutive weeks. After the completion of all behavioral tests, the brains of each group of mice were dissected and subjected to analysis. (**A**) ThS-stained insoluble Aβ plaque (left panel) and GFAP-stained (right panel) brain samples were shown for all treatment groups. Aβ plaques were stained with ThS: green; GFAP: red; and DAPI: blue. Merged images of Aβ plaques, GFAP, and DAPI staining of brain slices were also presented. (**B**) The total number of plaques in the hippocampus and cortex of all treatment groups were quantitated. (**C**) Quantitative analysis of GFAP immunofluorescence intensity. (**D**) Dot blot assay of brain lysates targeting the Aβ 1–16 (anti-Aβ:6E10), Aβ oligomers (anti-amyloidogenic protein oligomer: A11) and Aβ fibrils (anti-amyloid Fibril: mOC87) in the hippocampus and cortex of all treatment groups. (**E**–**F**) The expression level and quantification analysis of APP/GFAP/APP-β/BACE1 proteins in cortex lysates. (**G**–**H**) The expression level and quantification analysis of APP/GFAP/APP-β/BACE1 proteins in hippocampus lysates. All data were expressed as mean ± S.D, n = 6–8, ^#^P < 0.05, ^##^P ≤ 0.01, ^###^P ≤ 0.001, compared with WT group; *P < 0.05, **P ≤ 0.01, ***P ≤ 0.001, compared with APP/PS1 group; one-way ANOVA analysis. (For interpretation of the references to colour in this figure legend, the reader is referred to the Web version of this article.)

All the above APP/PS1 and WT mice were sacrificed after behavioral evaluation, and their brains were dissected and examined by histochemical and protein blot analysis. To investigate the effect of nasal administration of HYP on the deposition of Aβ plaques, brains from APP/PS1 mice were sectioned and stained with ThS [50] to visualize Aβ plaques (left panel of Fig. 5A). By quantitating the total number of plaques in both the cortex and hippocampus of APP/PS1 mice, Aβ plaques were observed in APP/PS1 mice at 28 weeks. Of noted, HYP treatment reduced the level of Aβ plaques in the cortex and hippocampus of APP/PS1 mice in a dose-dependent manner (Fig. 5B). Next, the immunohistochemical staining of the astrocytes marker, GFAP, representing astroglial activation and gliosis during neurodegeneration [51] was performed. Fig. 5A (right panel) and 5C showed that HYP administration decreased the level of GFAP in both the cortex and hippocampus of APP/PS1 mice when compared to APP/PS1 group, suggesting the neuro-protective role of HYP. There is evidence supporting that neurotoxicity induced by Aβ aggregation was attributed to Aβ oligomers rather than amyloid plaque itself [18,52]. To investigate whether HYP can prevent the formation of Aβ in the mouse brain, dot-blot analysis was adopted for the evaluation of both the cortex and hippocampus protein lysates using anti-β-amyloid (amino acid residue 1–16 of Aβ) (6E10), anti-amyloid oligomer (A11), and anti-amyloid fibril antibody (mOC87), respectively. Results showed that HYP reduced the level of both oligomeric and fibrillar form of Aβ, without altering the level of total Aβ (Fig. 5D). Western blot analysis further confirmed that HYP reduced the level of APP hydrolysis pathway-associated proteins such as BACE1 and APP-β, as well as astrocyte activation in both the cortex (Fig. 5E–F) and hippocampus (Fig. 5G–H) of APP/PS1 mice. The above data indicated that HYP alleviated the amount of Aβ plaques by reducing the formation of Aβ oligomers and fibrils in the brains of APP/PS1 mice.

### 3.6 HYP attenuated apoptotic cell death in the cortex and hippocampus of APP/PS1 mice

**Fig. 6:**
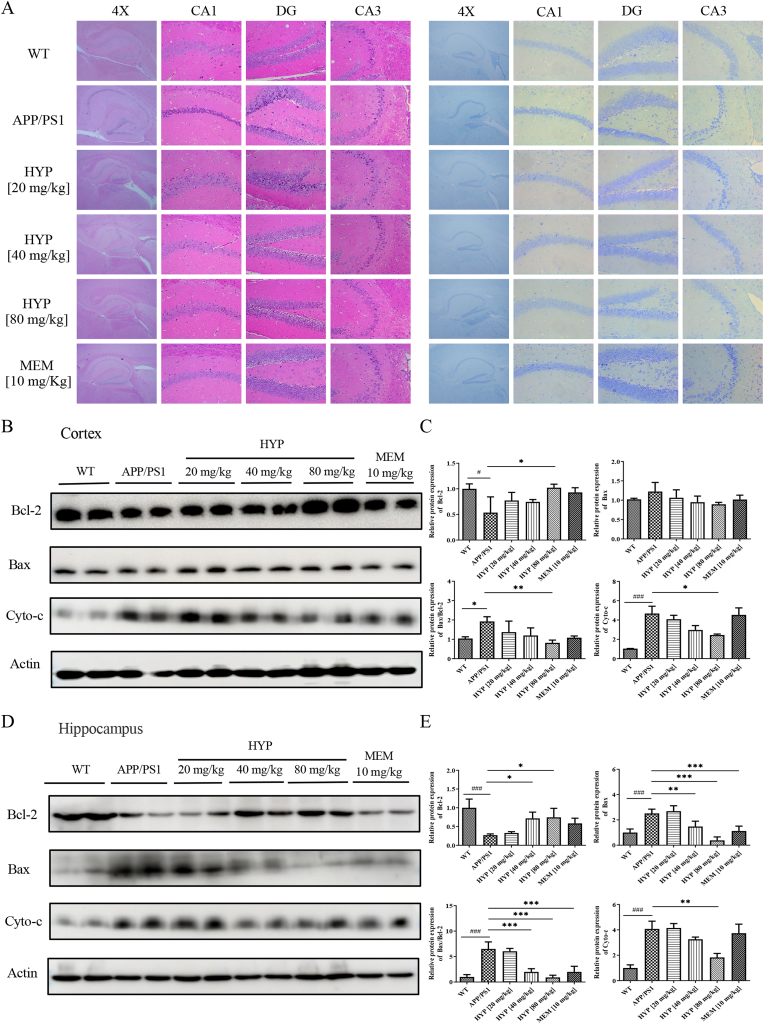
Effect of HYP in regulating Aβ-induced apoptosis in APP/PS1 mice model The brain samples of each group of mice were subjected to analysis after the completion of behavioral tests. (**A**) Representative H&E staining (left panel) and nissl staining (right panel) images of the CA1 regions, DG, and CA3 region of the hippocampus in the mice of all treatment groups. CA1, cornu ammonis region 1; CA3, cornu ammonis region 3; DG, dentate gyrus. (**B**–**C**). The expression level and quantification analysis of Bcl-2, Bax and Cyto-c proteins in cortex lysates. (**D**–**E**) The expression level and quantification analysis of Bcl-2, Bax and Cyto-c proteins in hippocampus lysates. All data were expressed as mean ± S.D, n = 6–8, ^#^P < 0.05, ^##^P ≤ 0.01, ^###^P ≤ 0.001, compared with WT group; *P < 0.05, **P ≤ 0.01, ***P ≤ 0.001, compared with APP/PS1 group; one-way ANOVA analysis.

One of the pathogenic markers of AD is the progressive loss of neuronal cell triggered by Aβ-induced apoptosis [53]. To examine the histological structure of cells in the brain tissue sample, Fig. 6A (left panel) showed the representative photomicrographs of HE staining in the hippocampus of different group of WT and APP/PS1 mice. In the hippocampal CA1, CA3, and DG region of the WT group, neuronal cells were intact and regular in morphology with dense and neat arrangement, and the nuclei were round or oval with clear borders and evenly distributed chromatin. However, disorganized neuronal cell morphology and unclear cytoplasmic boundaries of the nuclei, neuronal degeneration and nuclei atrophy were observed in the CA1, CA3, and DG areas of the APP/PS1 group. In contrast, neuronal cell morphological changes were minimal after nasal administration of HYP in APP/PS1 mice. Nissl staining widely adopted to evaluate morphology and pathology of neural tissue was used to examine the histopathological changes of the hippocampus. The results showed that the density of neuronal cells in the CA3 and DG regions was higher in the HYP treated group when compared to the APP/PS1 control group (right panel of Fig. 6A). In the WT group, neurons in the CA1, CA3 and DG regions of the hippocampus showed a large number of dense granule cells, pyramidal cells, and Nissl bodies. In contrast, neurons in the APP/PS1 group showed pathogenic morphologies such as sparse cell arrangement, swollen cell bodies, loss of integrity, cytoplasmic atrophy, and oval or triangular nuclei. The degeneration of neurons in the CA3 and DG regions of the hippocampus was significantly reduced after treatment of HYP. These results confirmed that HYP reduced the pathogenic changes of brain tissue. Bcl-2, an anti-apoptotic protein that inhibits the activation of the apoptotic cell death pathway; Bax, a pro-apoptotic member protein of Bcl-2; and cytochrome-c, a small molecule released from mitochondria into the cytoplasm during apoptosis. Therefore, the expression of the apoptosis-related protein Bcl-2, Bax and cytochrome-c (cyto-c) were evaluated in the cortex (Fig. 6B–C) and hippocampus (Fig. 6D–E) tissue. Western blot analysis showed a decrease in the level of both Bax and cyto-c, an increase in Bcl-2 expression, and a reduction in the Bax/Bcl-2 ratio after 8 weeks of HYP treatment when compared to the APP/PS1 untreated group. This evidence suggests HYP exerted *in vivo* neuroprotective effect through alleviating neuronal apoptosis in mice brain.

### 3.7 HYP rescued Aβ -induced neuronal cell death

**Fig. 7:**
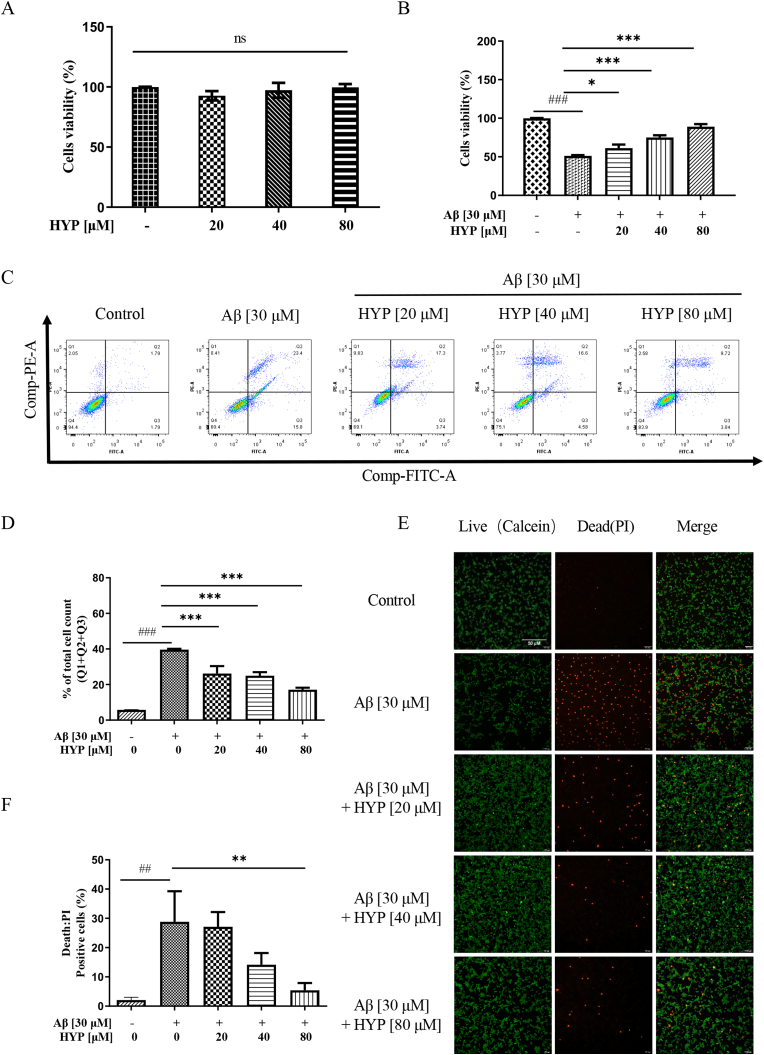
Effect of HYP in Aβ aggregate‐mediated cell death in HT22 cells (**A**) Cell viability of HT22 cells after 24 h of HYP (20–80 μM) treatment were measured by MTT assay (n.s., no significance, p > 0.05, when compared to untreated group, *t*-test). (**B**) Cell viability of HT22 cells after HYP (20–80 μM) treatment with or without aggregated Aβ co-treatment for 24 h. (**C**–**D**) The scatter plot and quantification of cell death were shown after flow cytometry analysis. The percentage of cell death at the indicated treatment conditions for 24 h were measured after annexin V- propidium iodide (PI) cell-staining. (**E**–**F**) Images of LIVE / DEAD cell analysis and the percentages of dead cells. Cells co-treated with Aβ and various concentrations of HYP for 24 h were stained by calcein-PI for 20 min, and then with representative fluorescence images captured. All data were presented as mean ± S.D, n = 3. *P < 0.05, **P ≤ 0.01, ***P ≤ 0.001, compared with the control (untreated) group; ^#^P < 0.05, ^##^P ≤ 0.01, ^###^P ≤ 0.001, compared with Aβ group, one-way ANOVA analysis.

To investigate how HYP reduces amyloid deposition and ameliorates apoptosis in the AD mice model, the effect of HYP on Aβ-induced cell death was evaluated. Firstly, the viability of HT22 cells treated with HYP (20–80 μM) was evaluated by MTT assay. As shown in Fig. 7A, no obvious cellular toxicity was observed within the tested concentration range of HYP alone (supplementary 1F and 2A-B). While aggregated Aβ (30 μM) induced cell death in HT22, co-treatment of HYP (20–80 μM) with aggregated Aβ (30 μM) increased the cell viability in a dose-dependent manner as confirmed by both MTT (Fig. 7B) and flow cytometry analysis (Fig. 7C–D). Furthermore, immunocytochemistry staining using calcein AM dye for live cells (green) and propidium iodide dye (red) for dead cells (Fig. 7E–F) further confirmed the increase of cell viability in aggregated Aβ-treated HT22 after HYP treatment, confirming the protective effect of HYP in Aβ-induced neuronal cell death *in vitro*.

### 3.8 HYP regulated calcium pathway-related genes

**Fig. 8:**
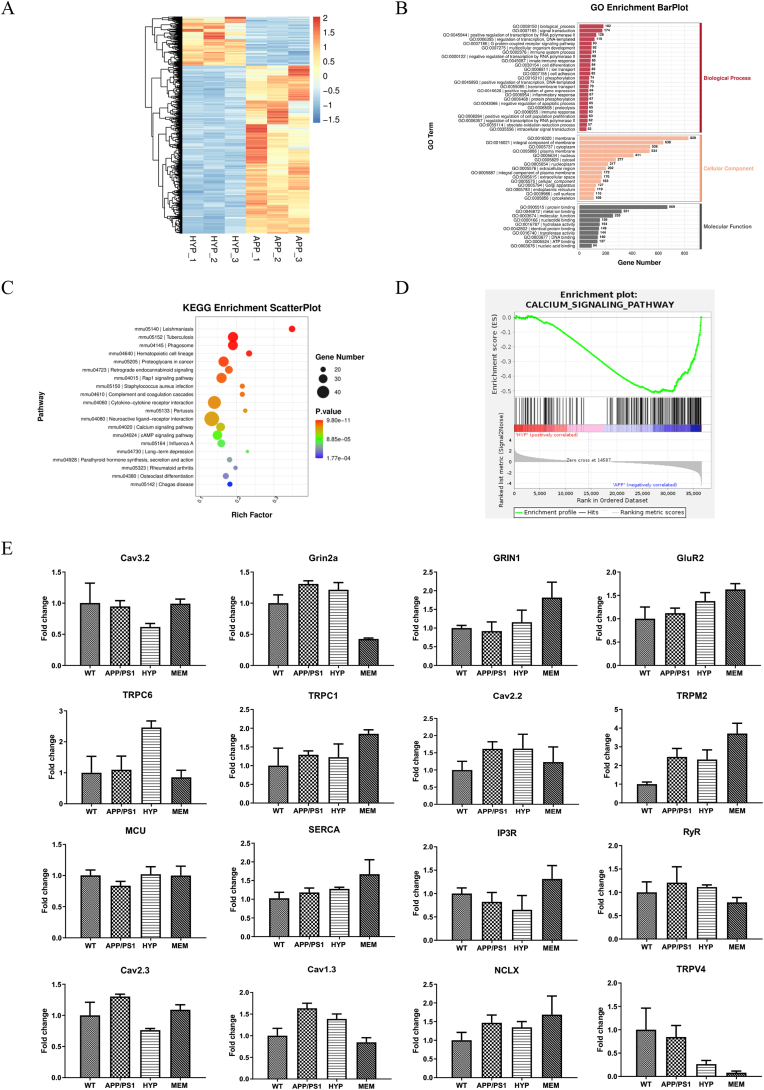
Gene pathway enrichment analysis by RNA sequencing **(A)** Hierarchical clustering dendrogram of gene expression: the horizontal axis at the bottom represents the name of samples and the vertical axis on the left side represents the degree of gene clustering. **(B)** GO enrichment analysis for key targets. Red indicates the biological process category; orange indicates the cellular component category and black indicates the molecular function category **(C)** KEGG pathway enrichment analysis of key targets (top 20 were listed); The X-axis shows the rich factor and the Y-axis shows the KEGG terms. **(D)** GSEA enrichment plots of all gene: calcium signal pathway. **(E)** RT-PCR validation of the calcium-related gene between HYP and APP/PS1 group. (For interpretation of the references to colour in this figure legend, the reader is referred to the Web version of this article.)

To investigate the molecular mechanism of HYP, initial data (36,668 raw reads) obtained from the RNA sequencing were performed. Hierarchical clustering analysis determining the correlations among samples through grouping at the gene level showed that the expression profiles were significantly different between HYP and APP/PS1 group (Fig. 8A). In GO classification, the unigenes were annotated in three major categories (Fig. 8B). The results showed that the differentially expressed genes were enriched in molecular function (MF), biological process (BP) and cellular component (CC). For the biological process category, most of the unigenes were matched to immune and signaling processes. For the cellular component category, most of the unigenes were assigned to cell and cell part. For the molecular function category, binding activity accounted for the largest proportion. The results of the KEGG analysis (Fig. 8C) demonstrated that differentially expressed mRNAs were mainly enriched in 20 biological pathways, including many AD-related metabolic pathways. Gene set enrichment analysis (GSEA) has been extensively applied to identify underlying pathways [54]. In Fig. 8D, GSEA analysis of calcium channels was performed in conjunction with the previous AD hypothesis. Next, the relative expression levels of the hub genes in the APP and HYP-treated mice groups were examined by RT-PCR analysis. The data showed that the mRNA expression levels of Cav2.3, Cav1.3, NCLX and TRPV4 were decreased in the HYP group compared to the APP/PS1 group, while opposite result was observed in MCU (Fig. 8E). This data suggested the possible involvement of calcium signaling in HYP treatment.

### 3.9 HYP attenuated Aβ -induced apoptosis via regulation of ryanodine receptor 2

**Fig. 9:**
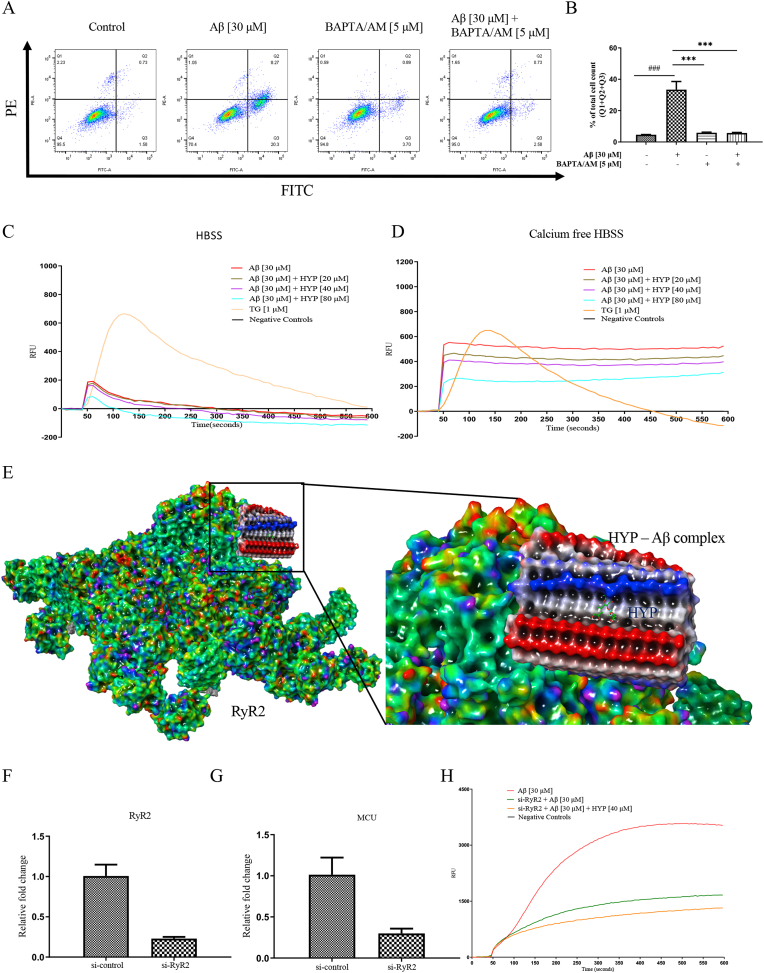
HYP regulated Aβ-induced Ca^2+^ dyshomeostasis via RyR2 (**A**–**B**) The scatter plot of flow cytometry showed the percentage of apoptotic cell death after HYP treatment in Aβ-induced cells. The percentage of cell death in Aβ-induced cells with or without BAPTA/AM co-treatment for 24 h was measured by flow cytometry analysis after annexin V- PI cell-staining. All data were expressed as mean ± S.D, n = 3. *P < 0.05, **P ≤ 0.01, ***P ≤ 0.001, compared with the untreated control group; ^#^P < 0.05, ^##^P ≤ 0.01, ^###^P ≤ 0.001, compared with Aβ group, one-way ANOVA analysis. (**C**–**D**) HT22 cells treated with 30 μM of Aβ dissolved in either calcium-containing or calcium-free HBSS dissolved with or without HYP (20–80 μM), or TG (positive control) were stained with FLIPR Calcium 6 Assay Kit, and then subjected to dynamic calcium measurement immediately by FLIPR Tetra High-Throughput Cellular Screening System. The mean value of RFU in the chart represented the dynamic cytosolic calcium levels. (**E**) The computation docking results of HYP - Aβ complex docked with RyR2 (PDB ID: 5GOA). (**F**) The mRNA level of RyR2 and (**G**) MCU in RyR2 siRNA transfected HT22 cells. (**H**) RyR2 was first knockdown in HT22 cells by RyR2 siRNA transfection, and then stained with FLIPR Calcium 6 Assay Kit after co-treatment of aggregated Aβ (30 μM) and HYP (40 μM). Dynamic calcium measurement by FLIPR Tetra High-Throughput Cellular Screening System was then performed, and the data from the chart was represented as the mean value of fluorescence level.

Although the underlying mechanisms of AD remains controversial, increasing evidence suggested that calcium (Ca^2+^) is an important factor for regulating the pathogenesis of AD [24]. Neurotoxicity induced by Aβ aggregates was also associated with intraneuronal Ca^2+^ dyshomeostasis [52]. In this study, fluorescent dye Fluo-3 was used to monitor the real-time level of Ca^2+^ (Supplementary 2C). With the significant increase of fluorescence intensity observed after the addition of Aβ in HT22 cells, the effect of HYP in Aβ -induced Ca^2+^ level change and toxicity were carried out. To begin, a cell-permeant chelator BAPTA/AM, was added to lower the intracellular level of intracellular Ca^2+^. As shown in Fig. 9A and B, BAPTA/AM was able to decrease the percentage of Aβ-induced cell death as measured by flow cytometry after annexin V-PI staining. Next, immediate dynamic Ca^2+^ level was measured by the FLIPR Tetra high-throughput cell screening system. TG, a non-competitive cell permeable inhibitor of calcium transport by sarco/ER Ca^2+^-ATPase (SERCAs), was adopted as the positive control for increasing the intracellular Ca^2+^ concentration. As shown in Fig. 9C, while a prompt increase in Ca^2+^ level was induced by TG or Aβ, the addition of HYP decreased the level of Ca^2+^ in a dose dependent manner. To further investigate how the Aβ-induced level of Ca^2+^ were regulated by HYP under an extra-cellular calcium-free condition, Aβ and HYP were co-treated with HT22 cells in calcium-free HBSS buffer. Results on dynamic Ca^2+^ monitoring showed that HYP still downregulated the Aβ -induced level of Ca^2+^ in a dose-dependent manner (Fig. 9D), indicating that both Aβ and HYP were able to regulate Ca^2+^ level via intracellular Ca^2+^ channels.

Neurons control calcium level via regulation on the intracellular calcium source such as the ER. Calcium release from the ER occurs through two type of calcium channels, the inositol-1,4,5-trisphosphate (Ins (1,4,5) P3) receptors (InsP3Rs) or the ryanodine receptors (RyRs) [55,56]. Therefore, computational docking targeting the RyR2 (PDB code: 5G0A) was performed as shown in Fig. 9E. The ligand interaction diagram indicated that the HYP- Aβ complex bind to RyR2 by chain A-D of Aβ (supplementary 2D). It is noteworthy that while HYP- Aβ complex bind well to RyR2 with a binding energy of −29.4 kcal/mol, HYP alone did not bind to RyR2. The role of RyR2 was further investigated through siRNA knockdown experiment. Fig. 9F showed that while the mRNA level of RyR2 was reduced by 77% in RyR2 siRNA knockdown cells, the mRNA level of mitochondrial calcium uniporter (MCU) that allows the cytosolic Ca^2+^ to enter the mitochondrial matrix [57] was also reduced by 70% (Fig. 9G). Furthermore, the dynamic Ca^2+^ level of Aβ and HYP treated RyR2 knockdown cells showed that, while Aβ-induced intracellular Ca^2+^ level was decreased compared with the untreated group, HYP restored the Aβ-induced dishomeostasis of Ca^2+^ in si-RyR2 HT22 cells (Fig. 9H) to a lesser extent. These results indicated that HYP attenuated Ca^2+^ dyshomeostasis induced by Aβ aggregates partially via regulation of RyR2.

### 3.10 HYP blocked Aβ -induced disruption of mitochondrial membrane potential and apoptosis in HT22 cells

**Fig. 10:**
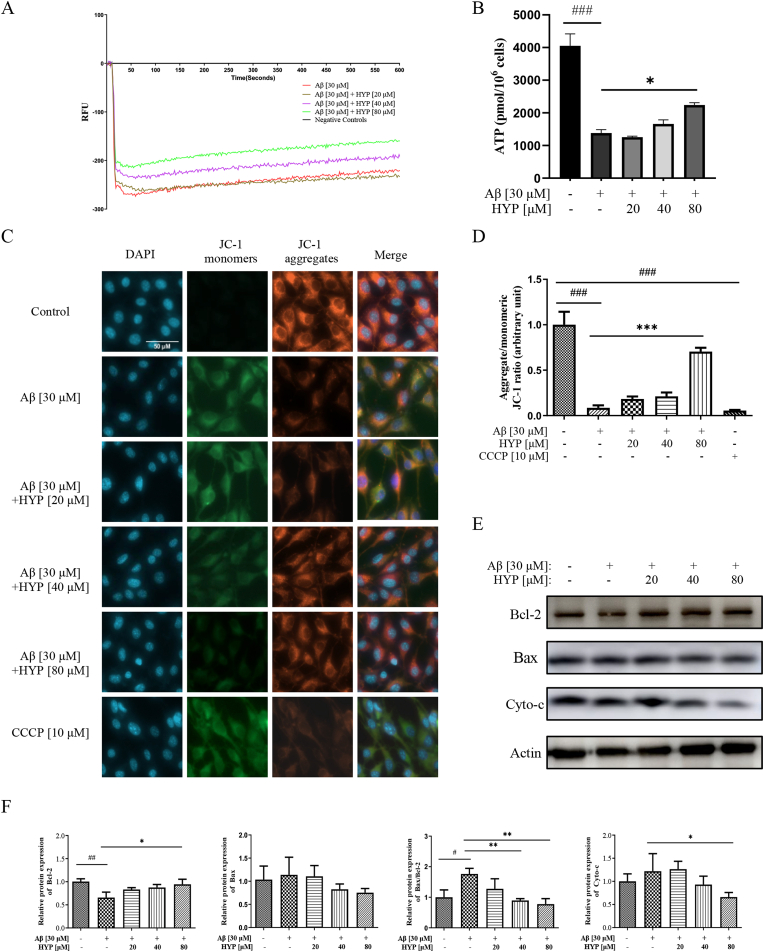
Regulation of HYP on Aβ-induced loss of mitochondrial membrane potential (**A**) The mitochondrial membrane potential of aggregated Aβ (30 μM) induced HT22 cells with or without HYP (20, 40, 80 μM) treatments were recorded. The mean value of the RFU represented the variation of mitochondrial membrane potential in 600 s after the indicated treatments. (**B**) ATP production (pmol/10^6^ cells) in aggregated Aβ HT22 cells with or without HYP (20, 40, 80 μM) treatment. (**C**) Representative images of HT22 cells stained by JC-1 for the quantitation of mitochondrial membrane potential. Cells treated with HYP with or without Aβ, or CCCP (positive control for mitochondrial membrane potential dissipation) were shown as indicated. The reduction of the red/green fluorescence intensity ratio represented mitochondrial depolarization. Scale bars = 50 μm. (**D**) Quantification of mitochondrial depolarization. (**E**–**F**) The expression level and quantification analysis of Bcl-2, Bax, and Cyto-c proteins in aggregated Aβ (30 μM) induced HT22 cells with or without HYP treatments. All data were expressed as mean ± S.D, n = 3. *P < 0.05, **P ≤ 0.01, ***P ≤ 0.001, compared with Aβ group; ^#^P < 0.05, ^##^P ≤ 0.01, ^###^P ≤ 0.001, compared with the control (untreated) group, one-way ANOVA analysis. (For interpretation of the references to colour in this figure legend, the reader is referred to the Web version of this article.)

Disruption of RyR2 regulation in the ER mediates the most important signal transduction cascade responses associated with AD [24]. RyR2 channels are implicated in many cellular functions, particularly mitochondrial metabolism [31]. As confirmed in previous section (Fig. 9G), the expression of mRNA for MCU was decreased in RyR2 knockdown cells. MCU is a highly selective Ca^2+^ channel located in the inner mitochondrial membrane responds to the Ca^2+^ release from the ER into the mitochondrial matrix [28], and increased mitochondrial Ca^2+^ influx lead to cell death. Therefore, the role of HYP in regulating Aβ-induced ER-mitochondrial Ca^2+^ homeostasis and apoptosis were further validated. Real time measurement of mitochondrial membrane potential in rhodamine 123-stained cells revealed that HYP attenuated the decrease of membrane potential triggered by Aβ (Fig. 10A). It was reported that mitochondrial calcium level affects cellular energy by activating oxidative metabolism, mitochondrial respiration and ATP synthesis [58]. The ATP content of HT22 cells was detected by LC-MS/MS and confirmed that HYP rescued the Aβ-induced decrease in mitochondrial ATP compared with the untreated group (Fig. 10B). To further verify the result, the membrane potential was stained with a more sensitive dye, JC-1 (Fig. 10C). JC-1 is a dual-emission mitochondrial membrane potential sensing dye, which is accumulated and aggregated in polarized (normal) mitochondria with excitation and emission in the red spectrum at ∼590 nm [59]. While uncoupler CCCP treatment lead to loss of membrane potential and prevented JC-1 from entering the mitochondrial membrane of cells, this kind of monomeric JC-1 was retained on the cell membrane and emitted green fluorescence. Fig. 10D showed the quantitated ratio of aggregated and monomeric JC-1 compared to the untreated group, suggesting HYP was able to rescue the loss of mitochondrial membrane potential induced by Aβ aggregates in a dose-dependent manner while HYP alone has no effect (Supplementary 2E-F). It is noteworthy that increased mitochondrial calcium influx can trigger apoptosis [60]. To further explore the protective role of HYP in Aβ -induced neuronal cell death, HT22 cells were co-treated with Aβ aggregates and HYP for 24 h. The expression level of selected apoptotic markers including Bcl-2, Bax and cyto-c were detected by Western blot (Fig. 10E). Consistently, the ratio of Bax / Bcl-2 was decreased with the reduction in the level of cyto-c (Fig. 10F) after HYP treatment, demonstrating that HYP may rescue cells from apoptosis via attenuating mitochondrial membrane potential loss in neuronal cells. Collectively, these results showed that HYP reduced Aβ-induced neuronal apoptosis through regulating the ER-mitochondrial Ca^2+^ level and its related signal transduction cascade.

## 4 Discussion

Alzheimer's disease is one of the most prevalent type of aged-related neurodegenerative diseases. Current available pharmacological treatments of AD are mainly targeting the pathogenic markers such as Aβ, tau and the AD-related inflammatory responses [61]. Many research works have confirmed Aβ has prominent downstream modulatory effects on tau phosphorylation, neurodegeneration and cognitive malfunction [62]. While the ATN framework involving Aβ and tau continues to be adopted as the pathogenic mechanism of AD [63], further downstream mechanisms involving the development of neurotoxicity in AD via disruption of Ca^2+^ homeostasis and mitochondrial dysfunction [64] were reported. In fact, the pathogenic mechanisms associated with Aβ toxicity are controversial [53]. While Aβ oligomer was reported as the most prevalent toxic forms of Aβ in AD [65,66], there is also substantial evidence that Aβ protofibrils also progressively induced apoptosis [67,68]. With the *in vitro* and *in vivo* study on Aβ aggregation and its toxic conformations [[69], [70], [71]], observation such as Aβ oligomers occur transiently in the pathway of protofibril formation [72] or Aβ in aqueous solution is mostly a mixture of interconverted conformations were reported. In this study, a mixture of Aβ aggregate form was used to set up the cellular Aβ toxicity assay. Interestingly, HYP reduced the protein levels of both Aβ oligomers and fibrils but has no effect on the levels of Aβ monomers, which may be beneficial for maintaining normal physiological functions of Aβ.

Consistent with the early manifestations of AD [30], Aβ aggregates were found to induce intracellular calcium dysregulation in our *in vitro* study. In the brain, RyR, SERCA, NMDA, VGCC receptors and SOEC were reported to play a pathogenic role in AD progression [[73], [74], [75], [76], [77]]. Among them, RyR2 localized in the hippocampus and cortex are shown to mediate the Ca^2+^ release and lead to the formation of Aβ plaque [78]. The Aβ can in turn activate RyR2 channels to promote the production of Aβ which further enhances RyR2 leakage and lead to a vicious cycle [79]. Aβ-induced Ca^2+^ overload leads to excess Ca^2+^ influx into mitochondria, which serves as the primary buffer system for calcium. This overload of calcium subsequently led to a decrease in membrane potential as well as cyto-c release [80]. In fact, Ca^2+^ uptake in the mitochondrial matrix is mediated by the highly conserved MCU complex [70]. Our current work has demonstrated that the knockdown of RyR2 is accompanied by decreased mRNA level of MCU expression, which may be responsible for the HYP-regulated Ca^2+^ homeostasis. Besides, with the evidence that the Ca^2+^ entry into the mitochondria is driven by the proton electrochemical gradient potential generated by the activity of the respiratory electron transport chain [81], the role of classical gatekeeper of the mitochondria calcium ion transport such as the voltage-dependent anion channel 1 (VDAC1) in the outer mitochondrial membrane (OMM) [82] still worth our further investigation. Previous studies have shown that limiting the opening time of RyR2 prevents Long-term Potential (LTP) impairment, learning and memory deficits, neuronal cell death and dendritic spine loss in 5xFAD mice [83,84]. One of the approach to inhibit RyR function is the use of dantrolene, which was reported to reduce AD deficits in various animal models [85], however, its clinical use is still limited due to its hepatotoxicity. Currently, approved AD drugs targeting the calcium signaling pathway include the L-type Ca^2+^ channel blocker nimodipine [86], and the NMDA open receptor blocker memantine [87] are available. In this study, HYP as an alternative natural compound specific for stabilizing RyR2 and targeting ER Ca^2+^, may be a promising agent for AD treatment by impeding the pathogenic cascade induced by Aβ and Ca^2+^ dyshomeostasis. However, over-reduction of RyR2 expression can also lead to detrimental effects on neuronal function [83,88], therefore, controlling the opening time rather than blocking the function and expression of RyR2 may be a safer way to modulate AD via RyR2. Previous reports have shown that HYP prevented protofibrillar Aβ (1–42)-induced brain endothelial cell damage [89] and increased learning and memory capacity by activating CP-AMPAR [90]. After 2 months of IN administration of HYP, our results confirmed that HYP reduced plaque deposition in the hippocampus of APP/PS1 mice and ameliorated neuronal cell damage in CA1 and CA3 regions. Although this beneficial effect can be attributed to HYP regulating the gene level of RyR2, more *in vivo* experiments are still needed for validation. In addition, ventricular injections and medullary delivery methods are relatively invasive and unsuitable for the treatment of chronic diseases in patients requiring long-term medication, therefore, studies on nasal delivery have been advocated in recent years. In fact, several low BBB permeability drugs have been shown to enter the CNS directly via the nasal route of administration [91], and IN administration leads to higher concentrations of HYP accumulation in the brain compared to IV administration. With the advantages of ease of handling and high blood-brain barrier permeability, therefore, HYP could be further developed as a nasal herbal formulation for the prevention and treatment of AD.

## Author contributions

Lin Lin Song: experimental design, data acquisition and analysis, manuscript preparation. Yuan Qing Qu, Hui Miao Wang: data acquisition and analysis, manuscript preparation. Xi Chen, Hui Xia Zhang and Wei Zhang: Measurement of ATP content. Li Qun Qu, Yun Xiao Yun: Pharmacokinetic experiments. Hang Hong Lo, Joyce Tsz Wai Chan: computational docking of Aβ, HYP and RyR2. Yong Pei Tang, Rui Long Zhang, Meng Han Liu, Cai Ren Wang, Jian Hui Wu: Animal behavior experiments. Betty Yuen Kwan Law, Vincent Kam Wai Wong: supervision and design of the project, data analysis and manuscript preparation.

## Declaration of competing interest

All authors declare no conflicts of interest.

## Data Availability

Data will be made available on request.
