# Supplementary material for: Hyperoside alleviates toxicity of β-amyloid via endoplasmic reticulum-mitochondrial calcium signal transduction cascade in APP/PS1 double transgenic Alzheimer's disease mice
Source: Redox Biol. 2023 Feb 14;61:102637. doi: 10.1016/j.redox.2023.102637 (PMC9975698; doi:10.1016/j.redox.2023.102637)

Supplementary 1

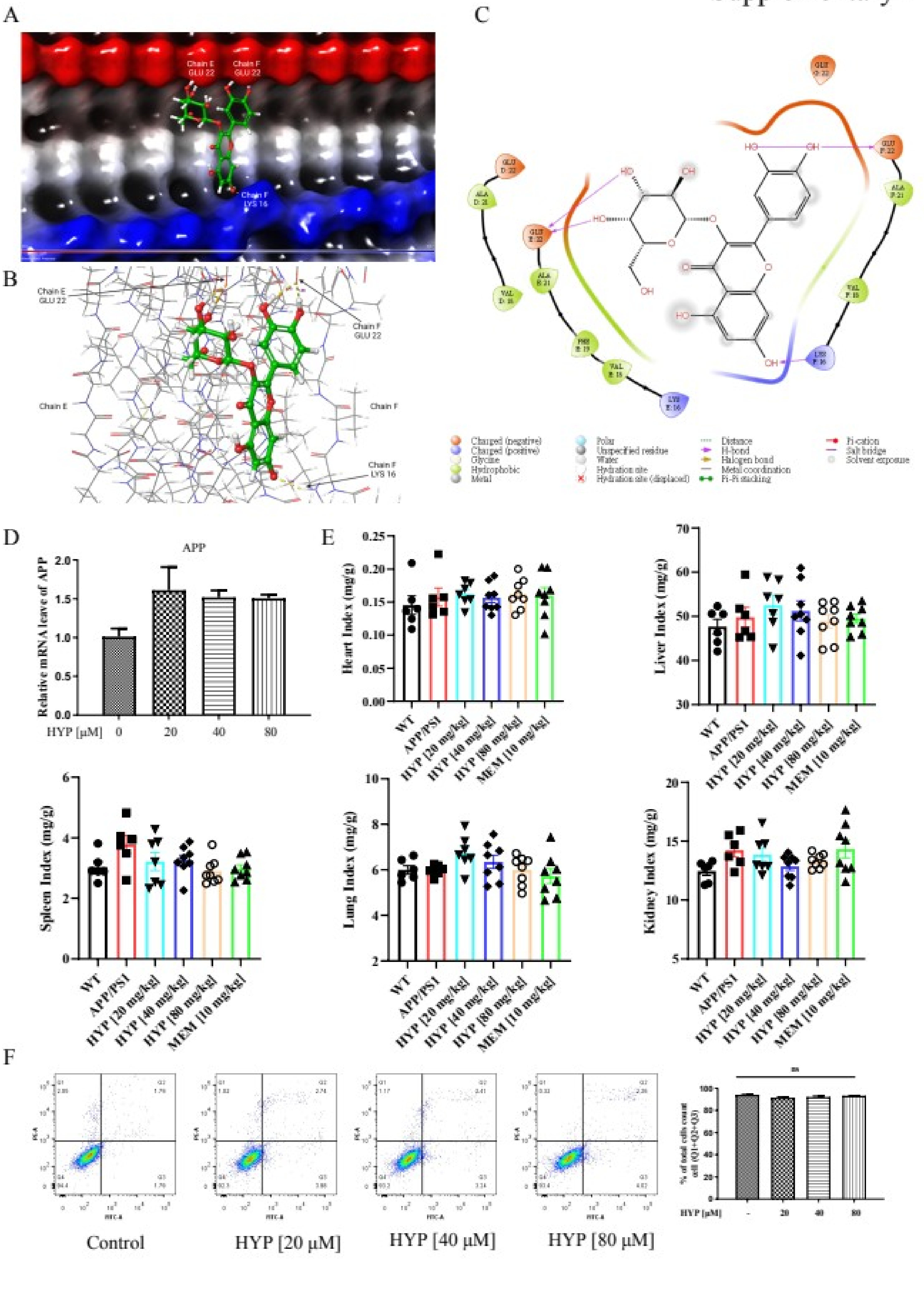

Supplementary 2

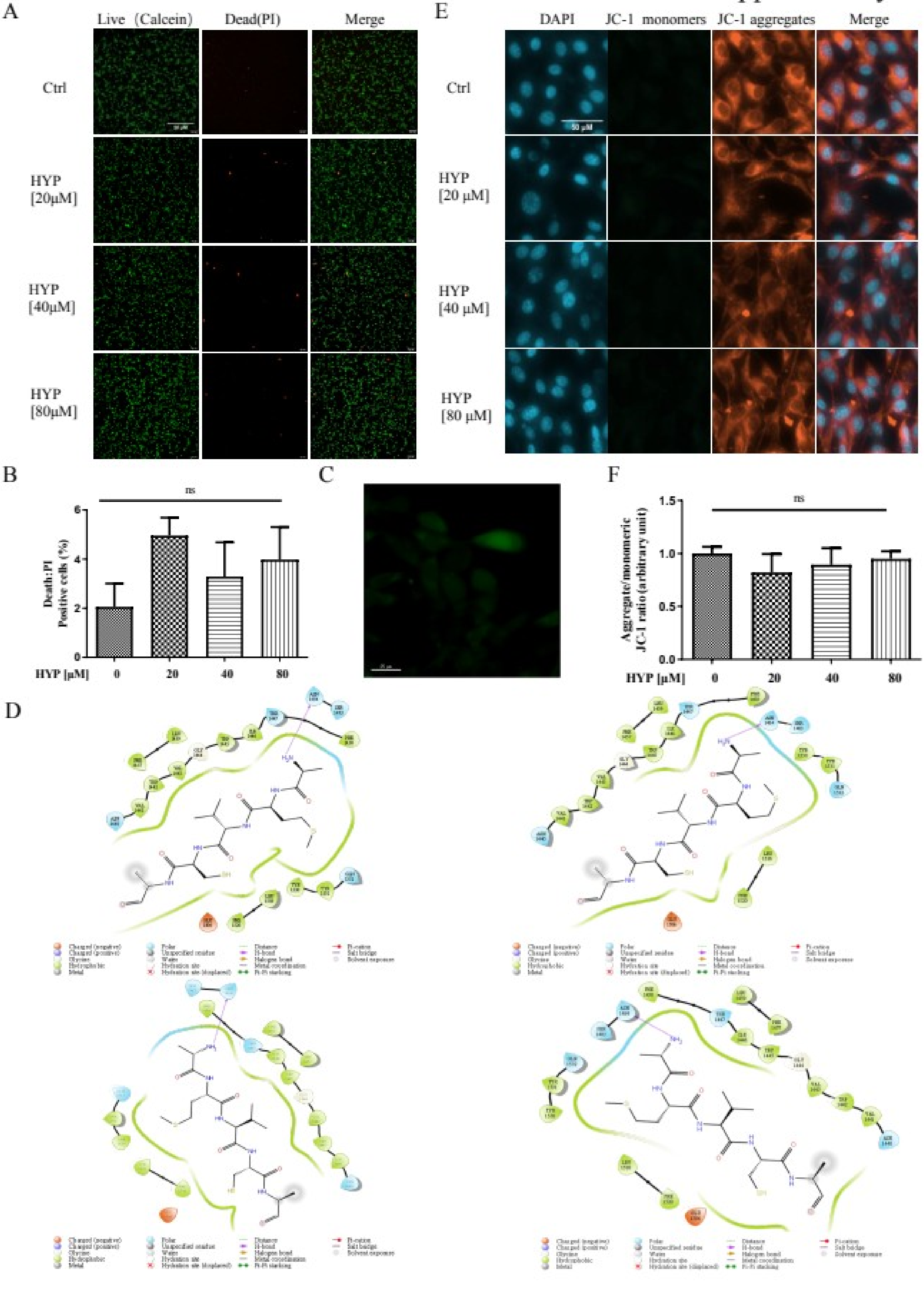

Supplement: Supplementary file 1 [file mmc2.docx]
